# Hydrophobic MFI‐Type Zeolites via Alkali‐Cation‐Induced Defect Healing: Implications for Adsorbent and Catalyst Design

**DOI:** 10.1002/adma.74455

**Published:** 2026-08-05

**Authors:** Christos Kanteler, Pau Ferri, Francisco Javier Escobar‐Bedia, Álvaro Mayoral, Alejandro Vidal‐Moya, Richard Kendra, Chen Lei, Miguel Palomino, Fernando Rey, Lukáš Grajciar, Christopher James Heard, Pedro Serna, Mercedes Boronat, Cristina Martínez, Manuel Moliner

**Affiliations:** ^1^ Instituto de Tecnología Química Consejo Superior de Investigaciones Científicas Universitat Politècnica de València València Spain; ^2^ Instituto de Nanociencia y Materiales de Aragón (INMA) CSIC‐Universidad de Zaragoza Zaragoza Spain; ^3^ Department of Physical and Macromolecular Chemistry Charles University Prague Czech Republic

**Keywords:** alkali cation‐mediated synthesis, defect engineering, hydrophobicity, Lewis acid catalysis, separation performance, zeolite

## Abstract

Here, we demonstrate that sub‐stoichiometric amounts of alkali cations (Na^+^ and K^+^) critically govern defect formation during the synthesis of Silicalite‐1 (MFI), enabling precise control over framework integrity and surface properties after calcination. Combining systematic synthesis studies with density functional theory (DFT) calculations and high‐resolution microscopy, we reveal a defect‐healing mechanism in which in situ generated NaOH or KOH species promote Si–O–Si bond rearrangement and enhance the mobility of Si(OH)_4_ units. This process facilitates the effective healing of T‐site vacancies, yielding highly ordered, defect‐free MFI frameworks. The resulting Silicalite‐1 exhibits markedly enhanced hydrophobicity and superior selectivity in butanol/water separation, underscoring the decisive role of defect control in modulating adsorption and interfacial properties. Importantly, these insights are successfully extended to the synthesis of defect‐free TS‐1, affording highly hydrophobic Lewis acid catalysts with improved activity and selectivity in the epoxidation of 1‐hexene. This environmentally friendly, straightforward, scalable approach offers a versatile pathway to produce defect‐free zeolites with precisely tuned physicochemical properties, enabling the development of advanced catalytic and separation materials, especially for applications involving water or polar compounds.

## Graphical abstract

Sub‐stoichiometric amounts of Na^+^ or K^+^ enhance defect healing during Silicalite‐1 (MFI) and TS‐1 calcination by promoting Si–O–Si annealing and healing framework vacancies. The resulting defect‐free zeolites are more hydrophobic and show improved butanol/water separation and improved activity and selectivity in the epoxidation of 1‐hexene, offering a simple route to tailor zeolite physicochemical properties for catalytic and separation applications.

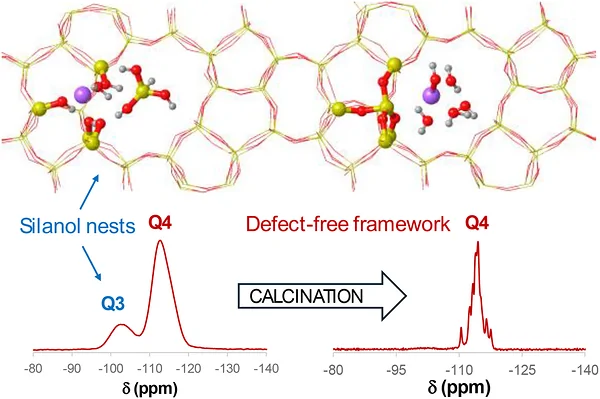

## 1 Introduction

The most practical synthetic procedure for achieving commercial application of zeolites relies on the use of highly alkaline conditions for mobilizing the inorganic silica and alumina species, avoiding the use of fluoride in their preparation [1]. However, this route results in the formation of microporous materials with remarkable internal silanol density (or structural defects) that are mostly created as a consequence of unbalanced positive charges from organic and/or inorganic cations during the zeolite crystallization and posterior calcination processes [2]. These internal defects or silanols, referred to as Si─OH, tend to be associated with connectivity defects and/or vacancy of framework heteroatoms that can result in the presence of so‐called silanol nests (normally represented by four neighboring Si–OH silanols, see Figure S1a) [2, 3, 4].

The presence of these silanols introduces non‐innocent acidity and increases the hydrophilicity of the material, thereby affecting its adsorption and catalytic properties for specific applications [2, 5, 6, 7, 8, 9]. For example, the presence of silanol groups in pure silica zeolites induces polar adsorption sites, reducing the selectivity in the separation of alcohols in alcohol/water mixtures [9, 10], thus limiting the production of bioalcohol in fermentative processes [11]. On the other hand, high internal silanol density is believed to promote catalyst deactivation in Brønsted‐catalyzed chemistries (by aluminosilicates), such as the methanol to hydrocarbon (MTH) reaction, as the coking formation rate is accelerated [6, 12, 13]. Their presence may also lead to undesired side reactions in processes catalyzed by Lewis acid zeolites (metallosilicates, including titano‐ or tin‐silicates, among others) as, for instance, the formation of diol‐type by‐products in olefin epoxidation reactions [5, 14, 15, 16]. Moreover, highly‐defective zeolite samples are, in general, less stable against hydrothermal treatments, since the degradation of high‐silica zeolites is typically originated at the framework defect sites [17, 18].

Methods to reduce the concentration of defects and silanols within the pores of zeolites can be classified into two main categories: (a) “one‐pot”, those that yield, very primarily, a defect‐free product upon crystallization of the zeolites from the corresponding precursors; (b) post‐synthesis approaches, where defects that exist in the calcined version of the materials are annealed through a subsequent thermo‐chemical treatment.

A long‐known one‐pot methodology to create highly hydrophobic defect‐free zeolite crystals is the fluoride‐mediated direct hydrothermal synthesis under neutral pH conditions [19, 20], where fluoride anions can be incorporated within the inner part of small secondary building units (SBUs) of the zeolite framework (such as in double 4‐membered rings, D4Rs), and then counterbalance the positive charges from the organic or inorganic cations trapped within the zeolite pores [19, 21]. The resultant defect‐free F‐mediated silicates provide enhanced organic/water separation selectivities (i.e., butanol/water) relative to traditional defective crystals prepared under alkaline conditions [10, 22, 23], whereas F‐mediated metallosilicate zeolite particles have been shown to be very active and more selective catalysts for different selective oxidation reactions [24, 25], as well as for biomass‐derived transformation in aqueous reaction media [26, 27]. However, the synthesis in fluoride media presents two main limitations: (1) zeolite crystals are remarkably larger than those prepared under alkaline conditions (within the micron‐scale) [20] and, (2) employing fluoride introduces serious environmental and safety restrictions due to the use and/or formation of HF during the zeolite preparation and/or activation [28].

In 2017, a different one‐pot methodology was reported to yield a silanol‐free tungsten‐containing Silicalite‐1 zeolite (MFI structure) in alkaline media under F‐free conditions [29]. The authors proposed that the flexible coordination environment of tungsten helps prevent the formation of silanol groups, as W(VI) species—specifically [O = W(‐OSi)_4_]—can coordinate with either four or two silicon atoms via oxygen bridges. This approach has also been applied to the synthesis of defect‐free Mo‐containing Silicalite‐1, where a similar coordination mode—[O = Mo(‐OSi)_4_]—was observed for molybdenum incorporation [30]. These zeolites are rare examples of defect‐free zeolites prepared by one‐pot synthesis methods under classical alkaline conditions. However, as expected, these approaches are highly restricted to the very limited number of metal species that exhibit such flexible coordination environments. Moreover, the use of metals such as Mo or W may introduce additional, application‐dependent undesired effects, underscoring the need for the development of a more general methodology.

Regarding the post‐synthetic approaches, recent liquid‐mediated post‐synthetic strategies have allowed to properly heal the defects in as‐prepared (a.p.) or treated zeolites, using silane precursors [31] or a combination of organic cations and fluoride species to fill or reconstruct the zeolite pores [18, 32]. Unfortunately, the use of silane precursors leads to considerable pore blockage, lowering the micropore volume [31], while the use of fluorinated species still raises safety and handling concerns for large‐scale industrial implementation.

Alternatively, silanol defects have been reduced in Silicalite‐1 (pure silica MFI structure) via gas‐phase treatments. Vicinal silanols, for example, can undergo dehydroxylation via condensation to form strained, doubly bridged structures upon simple high‐temperature thermal treatments (i.e., calcination above 600°C) [33], whereas more efficient defect healing in Silicalite‐1 can be achieved through high‐temperature steaming (i.e., ∼800°C) [8, 10], through an annealing mechanism that has not yet been fully elucidated. Despite the efficiency of this latter defect‐healing approach, the harsh conditions of the steaming process hinder its extrapolation to Al‐ or metal‐containing zeolitic materials, as these moieties suffer from additional stability issues.

Interestingly, early studies characterizing all‐silica Silicalite‐1 by ^29^Si MAS NMR reported that the presence of alkali cations (e.g., Na^+^ or K^+^, optimum content above 0.3 cations per unit cell of MFI—Si_96_O_192_) enables the healing of defects initially present in as‐prepared Silicalite‐1 during removal of the organic structure‐directing agent (OSDA), tetrapropylammonium (TPA), by direct calcination at 550°C [3]. The authors tentatively proposed that alkali cations promote the annealing of vicinal silanols (so‐called non‐bonding defects), but not silanol nests associated with T‐site vacancies [3]. However, the mechanism by which alkali cations induce defect annealing was not elucidated, nor were the physicochemical properties of the resulting defect‐free Silicalite‐1 materials investigated beyond ^29^Si MAS NMR spectroscopy. Furthermore, the applicability of this remarkably simple methodology to other systems, including metal‐containing zeolites relevant to adsorption and catalysis, was not assessed. As a result, this early observation was largely overlooked and remained unexplored in the subsequent literature.

Herein, we revisit the synthesis of Silicalite‐1 by introducing sub‐stoichiometric amounts of alkali cations (Na^+^ and K^+^) into the synthesis medium to investigate their role in controlling structural defects during simple calcination. This approach provides a new avenue for tuning the hydrophobicity of pure silica MFI zeolites, offering a sustainable alternative to traditional fluorinated media synthesis, which pose significant environmental and safety concerns and limit industrial applicability. By precisely controlling the sub‐stoichiometric incorporation of K^+^, our strategy enables a simple, scalable, and sustainable route to defect‐free, highly hydrophobic zeolites. A plausible defect annealing mechanism is proposed by density functional theory (DFT) computational simulation techniques, where the presence of sub‐stoichiometric NaOH or KOH species during the calcination treatment would catalyze Si─O─Si bond breaking and leaching of Si(OH)_4_ species responsible for healing T‐site vacancies. Moreover, fundamental learnings from the Silicalite‐1 system in the presence of sub‐stoichiometric alkali contents have allowed the efficient design of defect‐free TS‐1 (titanosilicate form of MFI) materials, leading to OH‐media synthesized hydrophobic Lewis acid catalysts. The defect‐free Silicalite‐1 and TS‐1 materials were evaluated in processes of significant industrial relevance. Specifically, Silicalite‐1 was investigated for the selective adsorption of butanol from butanol–water mixtures, a system of interest in biofuel production via fermentation processes. In parallel, TS‐1 was assessed as a selective catalyst in the epoxidation of 1‐hexene using H_2_O_2_ as oxidant. We believe that this very simple post‐synthetic approach using sub‐stoichiometric amounts of alkali cations can be generalized to design a large variety of defect‐free zeolites with fine‐tuned properties for novel and challenging catalytic processes, for instance, in biomass‐derived processes requiring tolerance or resistance to water and other polar compounds (e.g., oxygenates).

## 2 Results

### 2.1 Synthesis and Characterization of Silicalite‐1 Materials: Influence of Alkali Cations in Sub‐Stoichiometric Contents on the Physico‐Chemical Properties

Syntheses of the Silicalite‐1 materials were carried out under constant conditions (gel compositions and crystallization temperature) but incorporating sub‐stoichiometric sodium or potassium contents in the gels (see synthesis details for S1‐Na and S1‐K, respectively, in the Experimental Section). To adequately evaluate the role of the alkali cations, we also prepared the alkali‐free Silicalite‐1 material under analogous conditions (see synthesis details for S1 in the Experimental Section).

After 3 days of crystallization at 175°C, the three materials present the characteristic PXRD pattern of the MFI structure (see Figure 1a) and similar particle sizes of ∼200‐300 nm as revealed by FESEM images (see Figure 1c). All three Silicalite‐1 materials incorporate ∼4 TPA molecules per MFI unit cell, as deduced from the weight loss measured by thermogravimetric analyses in air between 200 and 650°C (see Figure S2a and Table 1), the N and C contents determined by elemental analyses (∼0.88–0.89 and 9.19–9.20 wt.%, respectively, see Table S1), and the fact that the MFI unit cell contains 96 T atoms in framework positions.

**FIGURE 1:**
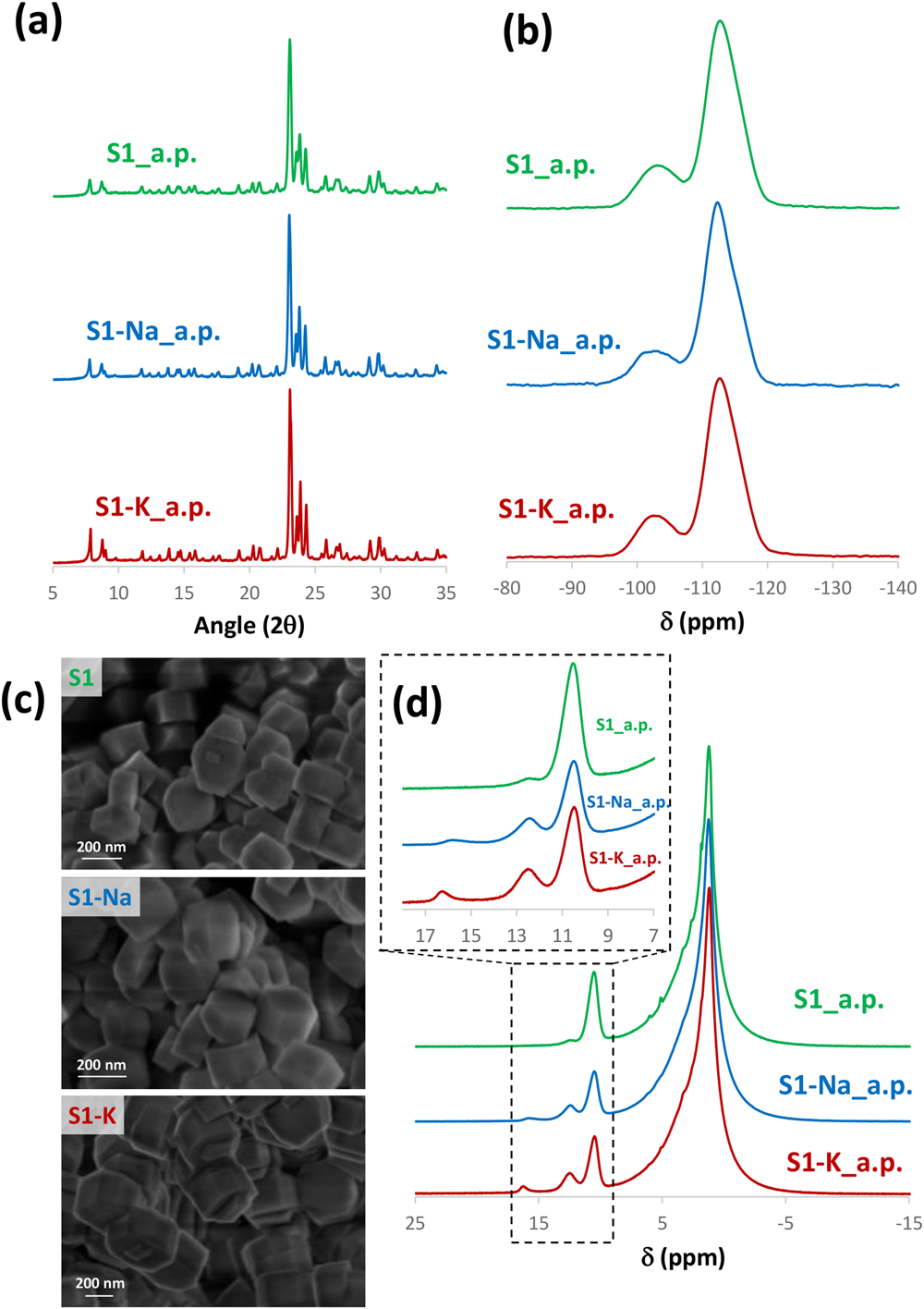
Characterization of the as‐prepared Silicalite‐1 materials prepared in absence (S1_a.p.) or presence of sub‐stoichiometric content of inorganic cations (Na or K, S1‐Na_a.p. or S1‐K_a.p., respectively): (a) PXRD patterns, (b) ^29^Si MAS NMR spectra, (c) FESEM images and (d) ^1^H MAS NMR spectra.

**TABLE 1:** ICP and TG of the different as‐prepared MFI materials (Silicalite‐1 and Ti‐Silicalite‐1) prepared in absence or presence of sub‐stoichiometric content of inorganic cations (Na or K), and their textural properties after calcination and removal of the inorganic cations (if required).

Sample	Si/M	Cat	Ratio	Cat/u.c.^a^	Weight loss (%)^b^	TPA/u.c.^b^	S_BET_ (m^2^/g)	S_ext_ (m^2^/g)	V_micro_ (cm^3^/g)
	***Si***		***Cat/Si***						
S1	∞	—	0.000	0.00	12.3	4.2	375	22	0.17
S1‐Na	∞	Na	0.009	0.85	13.2	3.9	342	28	0.16
S1‐K	∞	K	0.011	1.02	12.9	4.1	355	31	0.17
	***Si/Ti***		***K/(Si+Ti)***						
TS1	87	—	0.000	0.00	12.9	4.1	373	23	0.17
TS1‐K	80	K	0.012	1.15	12.7	4.1	390	24	0.17

^a^ u.c.: unit cell of MFI (96 T atoms).

^b^ Calculated from the thermogravimetric analysis (TGA) between 200 and 650°C in the as‐prepared samples.

Interestingly, chemical analyses by ICP confirm the sub‐stoichiometric incorporation of Na and K within the crystalline S1‐Na and S1‐K materials, with a cation/Si molar ratio close to ∼0.01 in both cases, corresponding to ∼1 inorganic cation per MFI unit cell (see Table 1). Elemental analysis by STEM‐EDS mapping of the as‐prepared S1‐K sample indicates a homogeneous distribution of K throughout the zeolite crystals (see Figure 2b‐bottom, with the corresponding line profile provided in Figure S3b). The required charge balance for ∼4 TPA molecules per MFI unit cell in the as‐prepared S1 material, as well as for ∼4 TPA molecules and ∼1 alkali cation per MFI unit cell in the as‐prepared S1‐Na and S1‐K materials, permits envisioning the formation of highly defective Silicalite‐1 crystals to compensate the positive charges of the occluded organic and inorganic cations [34]. To gather experimental evidence, the three as‐prepared Silicalite‐1 materials were further investigated by ^29^Si MAS NMR (see Figure 1b), which reveals similar large Q_3_ signals centered at ‐102 ppm—and assigned to Si─OH species—in all cases (see S1_a.p., S1‐Na_a.p., and S1‐K_a.p.), and a very broad Q_4_ signal between −110 and −120 ppm, assigned to the tetrahedrally coordinated Si species in different crystallographic T sites. This undefined, broad Q_4_ signal is characteristic of highly defective MFI frameworks, typical of alkaline syntheses. A semi‐quantitative integration of the Q_3_ and Q_4_ signals suggests the existence of ∼20% Si─OH species in the three as‐prepared materials, compatible with, at least, the presence of 4 T‐vacancies in the MFI unit cell, as (Si─OH)_3─_Si─O^−^ silanol nests associated with the TPA and/or alkali cations (see Figure S1a).

**FIGURE 2:**
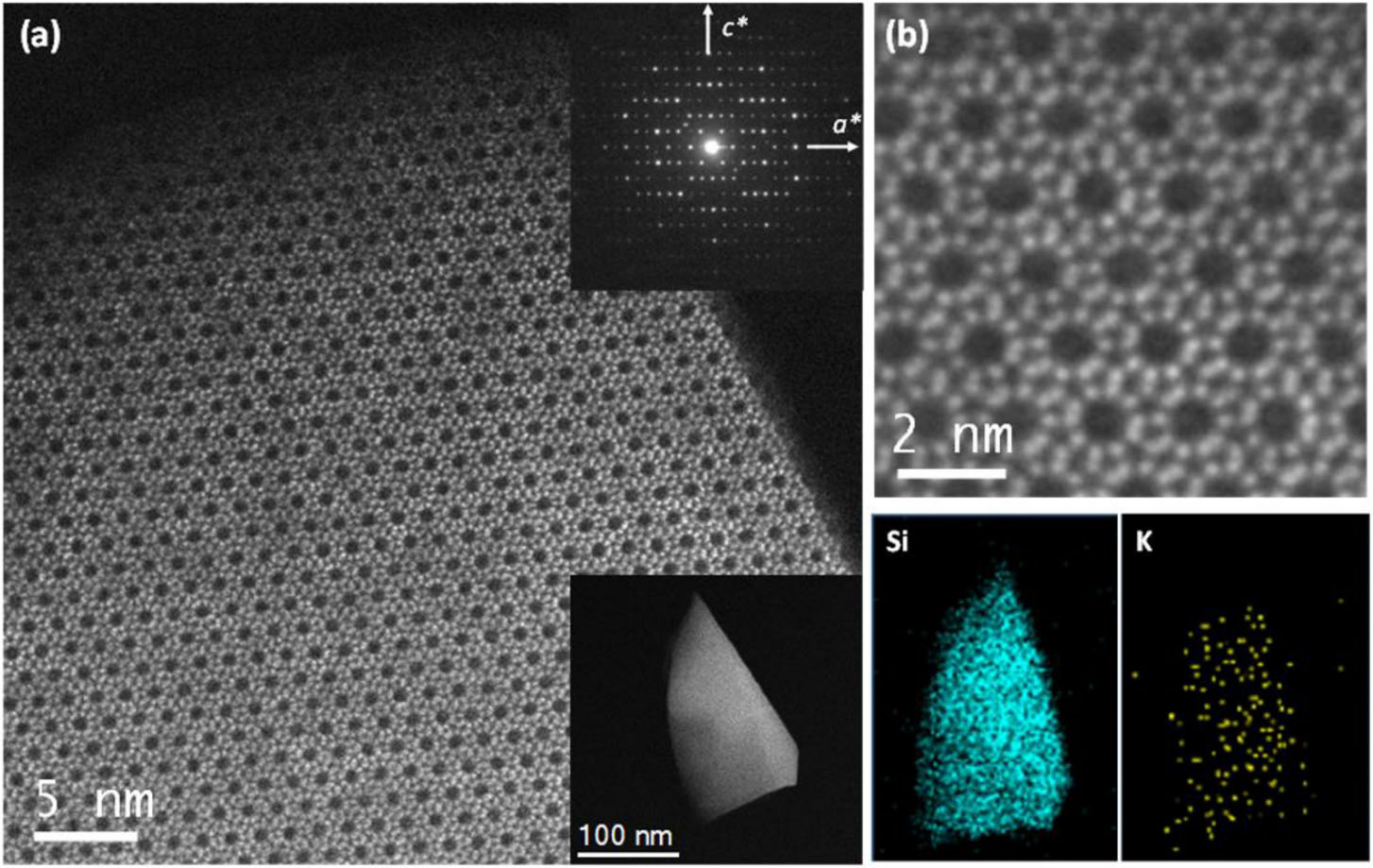
Cs‐corrected STEM‐ADF analysis of S1‐K_a.p. (a) High‐magnification image along the [010] zone axis with the FFT and low magnification image of the entire particle on the inset. (b) Atomic resolution data (top) and chemical maps (bottom) where Si and K appear in blue and yellow, respectively.

To better clarify the nature of the silanol species as well as to improve their quantification, the three as‐prepared Silicalite‐1 samples were also characterized by ^1^H MAS NMR spectroscopy. The three materials present a similar large asymmetric main signal centered at 2 ppm (see Figure 1d), assigned to the 112 protons of the methyl and methylene groups in the ∼4 TPA molecules per MFI unit cell (see Table 1). Additionally, the three materials also show signals at 10.2 and 12.4 ppm (see Figure 1d), both assigned to defective sites containing a Si‐vacancy (see Figure S1a). Interestingly, a detailed integration of these two signals accounts for ∼12 protons associated to the presence of four (SiOH)_3_−SiO^−^ clusters (see Figure S1a) per MFI unit cell that would balance the positive charges from the 4 TPA cations.

Finally, the as‐prepared S1‐Na and S1‐K samples also reveal very small signals centered at 15.5 and 16.4 ppm, respectively (see Figure 1d‐inset), whose integration corresponds to ∼1 proton per MFI unit cell. Since the content of the alkali cation is ∼1 per unit cell in both cases, we propose the formation of SiOH─SiO^−^ connectivity defects compensated by either Na or K to maintain the charge neutrality when sub‐stochiometric contents of alkali cations are present (see Figure S1b).

Interestingly, significant differences in the defectiveness of the three Silicalite‐1 materials are observed after a standard calcination treatment, as revealed by ^29^Si MAS NMR (see Figure 3a). On the one hand, the alkali‐free S1 sample maintains ∼7% of Q_3_ silanol species after calcination in air to remove the organic template, and maintains the Q_4_ signal broad, in agreement with a still very defective structure (see S1_cal in Figure 3a). On the other hand, a substantial decrease in the Q_3_ signal intensity is observed after calcination when sub‐stoichiometric amounts of alkali cations (∼1 alkali cation per unit cell) are present in the Silicalite‐1 materials, concomitant with a sharpening of Q_4_ signals as the silanol content decreases (see S1‐Na_cal and S1‐K_cal in Figure 3a). Particularly noteworthy is the almost complete removal of the Q_3_ signal in the calcined S1‐K sample, as well as the high‐definition appearance of several Q_4_ signals (see S1‐K_cal in Figure 3a), reminiscent of defect‐free Silicalite‐1 samples synthesized in fluoride media [3, 20, 32]. This result could be regarded as a significant breakthrough, enabling the straightforward one‐pot synthesis of low‐defect (or defect‐free) zeolite crystals by introducing an ultra‐low amount of alkali cations into the synthesis gels followed by conventional calcination, thereby avoiding hazardous F‐based methods [20, 24] or intricate post‐synthetic liquid‐phase treatments [18, 31, 32]. It can also be stated that MFI‐type zeolite can be synthesized with reduced crystal size, which is advantageous for improving molecular diffusion. The attainment of such small crystal dimensions is typically challenging when employing conventional fluoride‐mediated synthesis routes for MFI zeolites.

**FIGURE 3:**
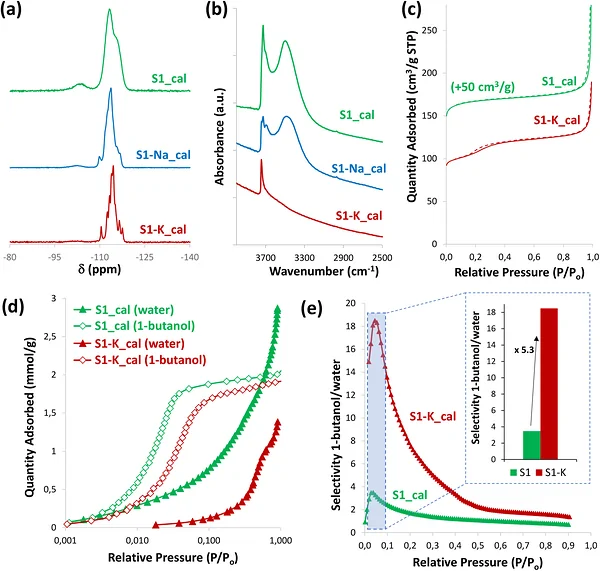
Characterization of the calcined Silicalite‐1 materials prepared in absence (S1_cal) or presence of sub‐stoichiometric content of inorganic cations (Na or K, S1‐Na_cal or S1‐K_cal, respectively)) after removing the inorganic cations (if required). (a) ^29^Si MAS NMR spectra, (b) IR spectra, (c), N_2_ adsorption/desorption isotherms at −196°C, (d) water and 1‐butanol adsorption isotherms at 25°C, and (e) 1‐butanol/water adsorption selectivities.

To fully confirm the disappearance of the framework defects in the sample prepared with sub‐stoichiometric K content in the synthesis gels, the resultant calcined S1‐K after additionally removing the K cations (see Experimental Section for details) was characterized by FTIR spectroscopy and compared with the calcined S1 directly prepared under alkali cations‐free conditions. The S1_cal sample directly prepared under alkali cations‐free conditions clearly shows the presence of two signals appearing at ∼3740 and ∼3500 cm^−1^, which correspond to terminal silanol species on the external surface and inner silanol nests, respectively (see S1_cal in Figure 3b). In contrast, the calcined and exchanged S1‐K sample only shows the presence of the terminal silanol species on the external surface at ∼3740 cm^−1^, but the signal centered at ∼3500 cm^−1^ associated to vacancy‐mediated inner silanol nests has fully disappeared (see S1‐K_cal in Figure 3b), in good agreement with the results achieved by ^29^Si MAS NMR spectroscopy.

The textural properties of the Silicalite‐1 materials, S1 and S1‐K, have been also evaluated by N_2_ adsorption/desorption at 77 K. As it could be expected for materials with analogous crystallinity and particle sizes, as inferred from the respective PXRD patterns (see Figure 1a) and SEM images (see Figure 1c), the measured BET surface areas (∼340–375 m^2^/g, see Table 1) and micropore volumes (∼0.16–0.17 cm^3^/g, see Table 1) are similar in both cases. However, noticeable differences are observed when comparing their N_2_ adsorption/desorption isotherms (see Figure 3c). The calcined S1‐K sample presents a step in the range 0.2 < relative pressure < 0.3, which is absent in the N_2_ adsorption isotherm of the calcined S1 sample (see Figure 3c). This observation has been reported in the literature when comparing Silicalites‐1 with different symmetries, orthorhombic or monoclinic, which normally corresponds to materials with a large or few number of OH concentration in the structures, respectively [35]. The enhanced N_2_ uptake in the orthorhombic samples, as well as the absence of the sharp step in the adsorption isotherms, have been assigned to the presence of silanol nests due to T‐vacancies, resulting in hydroxylated nanovoids [35].

### 2.2 Effect of Alkali Cations on the Annealing Mechanism of Silanol‐Nest Defects

Recently, Wakihara et al. have proposed a self‐defect cooperative healing mechanism that combines tetraethylammonium hydroxide and ammonium fluoride to facilitate the inner migration of silicate species in the liquid phase, leading to condensation of silanol nests in high‐silica Beta zeolite [18]. Additionally, Li et al. have recently been able to observe by using advanced electron microscopy (EM) that the adjacent silicon species to some former Si‐vacancies in MSE‐type zeolites would be leached and create a small mesopore during steam treatments, and these leached Si(OH)_4_ species would heal other isolated vacancies after migration through MSE pores [36].

Considering these recent observations on the “in situ” annealing mechanisms by creation of small voids or mesopores within zeolite crystals, we can hypothesize here that the presence of sub‐stoichiometric inorganic cations (Na^+^ or K^+^) in the as‐prepared Silicalite‐1 crystals could “locally” promote the presence of NaOH or KOH species during the calcination treatment, which are are well‐known species able to break and/or reorganize Si─O─Si bonds. Thus, these ultra‐low and catalytic contents of alkali cations within the as‐prepared Silicalite‐1 crystals would trigger the annealing process of the large amount of former framework vacancies in the as‐prepared material by an extremely simple calcination process.

High‐resolution imaging of the zeolite frameworks of the S1‐K samples, in both their as‐prepared and calcined forms, was performed by aberration‐corrected (Cs‐corrected) STEM‐ADF analysis. The results clearly confirm the highly crystalline nature of S1‐K_a.p. (see Figure 2a,b) and S1‐K_cal (see Figure 4a,b), with no a priori discernible structural differences between the calcined and as‐prepared materials, in agreement with the PXRD data. Although a substantial concentration of vacancies is expected in the as‐prepared S1‐K sample, their direct visualization remains intrinsically challenging. STEM images correspond to two‐dimensional projections of a three‐dimensional structure, and the projected lattice contrast arising from atomic columns located above and/or below the focal plane may obscure local features associated with missing structural units.

**FIGURE 4:**
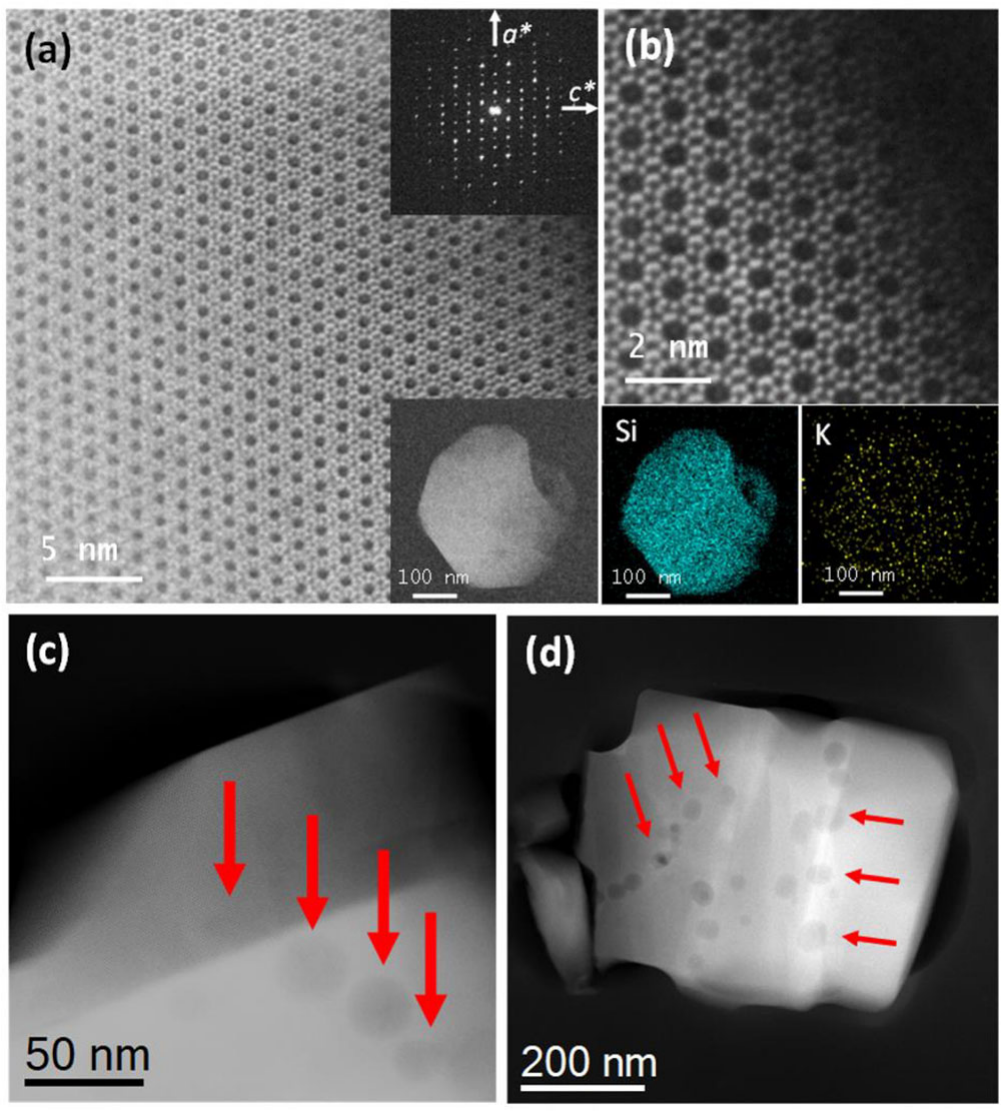
Cs‐corrected STEM‐ADF analysis of the S1‐K_cal. (a) High‐magnification image along the [010] with the FFT and low‐magnification image in the inset. (b) Atomic structural visualization of the edge of the crystal together with the chemical maps (Si in blue and K in yellow). (c,d) Low‐magnification images that reveal the formation of larger voids (red arrows).

While atomic‐resolution images do not reveal significant differences between the two samples, medium‐magnification observations highlight notable contrasts. The as‐synthesized S1‐K_a.p. material exhibits subtle contrast variations, including locally blurred regions that may be attributed to vacancy‐rich domains and, consequently, to local thickness fluctuations within the crystal (see Figure 5a, red arrows). In contrast, the S1‐K_cal sample displays a more homogeneous contrast, with the absence of blurred regions, consistent with vacancy filling upon calcination (see Figure 5b). Furthermore, low‐magnification images of individual particles do not show distinctive morphological features in the as‐prepared material (see Figure S3a). However, in the calcined sample, large internal voids (> 20 nm, see red arrows in Figure 4c,d), which are absent in the as‐made material, become evident. This observation suggests partial framework dissolution during calcination, in a manner analogous to the healing‐reorganization mechanism in liquid phase proposed by Wakihara et al. using TEAOH and NH_4_F [18].

**FIGURE 5:**
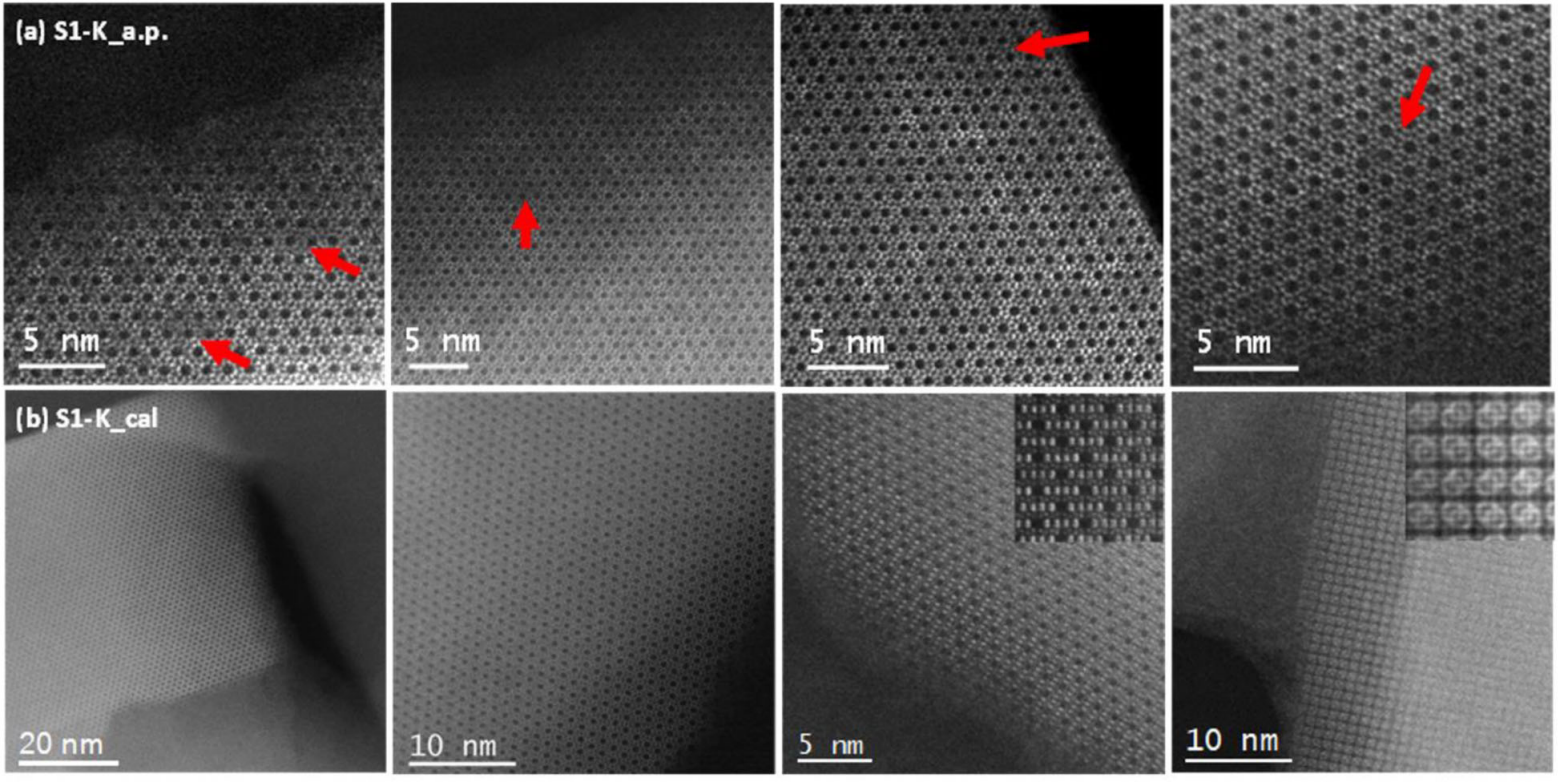
(a) Cs‐corrected STEM‐ADF images of S1‐K_a.p., where the red arrows point at domains that exhibit lower contrast associated with thickness variations due to the existence of vacancies. (b) Cs‐corrected STEM‐ADF images of S1‐K_cal along different zone axis displaying homogeneous contrast along the framework. Atomic resolution data is depicted in the inset of some images.

To elucidate the role of inorganic alkali cations in the defect‐healing mechanism, we performed a computational analysis of the cooperative annealing of a vacancy at a T3 crystallographic position in MFI, consistent with previous ^29^Si MAS NMR and PXRD studies [37]. The mechanism involves the progressive condensation of a migrating Si(OH)_4_ monomer along an MFI straight channel in the presence of water, following the full Q0→Q4 sequence. Calculations were carried out for Na^+^ and K^+^, and extended to Li^+^ to systematically probe the effect of cation size and charge density.

The starting configuration (Q0) consists of a silanol nest formed by three framework OH groups (with oxygens labeled O2, O3, O4) and one OM group (O1; M = Li, Na, or K), together with a coordinated water molecule and one Si(OH)_4_ monomer (with oxygens labelled O5–O8) diffusing along the straight channel (see Figure 6). In this post‐calcination‐like state, the alkali cation initially coordinates to O1 bearing a negative charge. Defect healing proceeds through a repetitive sequence of mechanistically analogous steps: pentacoordinated condensation transition states (TSQn_Qn+1), followed by proton‐transfer steps (TSHS) mediated by water. In each condensation step, a framework oxygen (O1 to O4) binds axially to the silicon atom in Si(OH)_4_, while an opposing Si–OH bond (O5 to O8) elongates, enabling proton transfer and water formation. Charge redistribution is achieved through cation migration between the partially condensed silicate and neighboring framework oxygens within the silanol nest, stabilizing charged intermediates and driving the stepwise evolution from Q0 to Q3. Final healing to the fully condensed Q4 state occurs via a distinct proton‐transfer transition state (TSQ3_Q4) between O4 and O5 mediated by a water molecule, yielding a healed framework site and an MOH species, whose hydroxyl group remains axially coordinated to the silicon atom, completing vacancy sealing (see Figures 6b and S4–S9).

**FIGURE 6:**
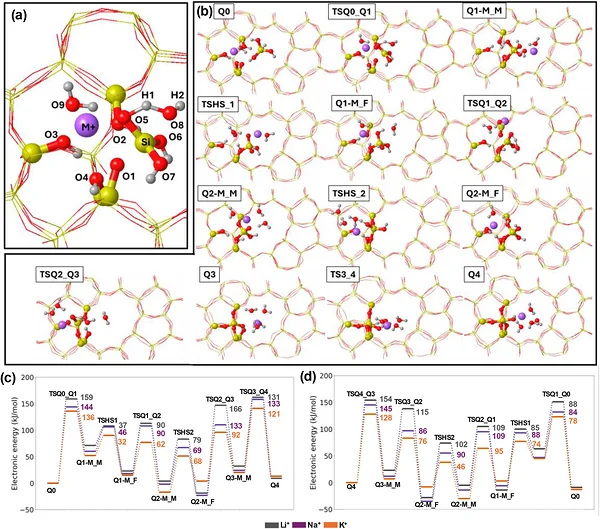
(a) Atom labeling scheme used to describe the annealing mechanism, illustrated for the TSQ0_Q1 structure. (b) Schematic representation of the annealing mechanism studied. Oxygen, silicon, hydrogen, and alkali cations (M^+^) are shown in red, yellow, white, and purple, respectively. M_M and M_F denote configurations in which the alkali cation is coordinated either to an oxygen atom of the Si(OH)_4_ monomer (M_M) or to a framework oxygen (M_F). (c,d) Relative energy paths in kJ/mol for healing Q0→Q4 (c) and cleaving Q4→Q0 (d) of a vacancy site. Gray, purple, and orange correspond to Li^+^, Na^+^, and K^+^, respectively. Full data for reproducing this figure is provided in Table S2. Numbers shown in each color next to the barrier plateaus indicate the stepwise barrier for the corresponding alkali cation (see Table S3).

The calculated activation barriers for condensation TSQn_Qn+1 steps follow a consistent trend across the alkali cation series: Li^+^ exhibits the highest barriers, Na^+^ intermediate values, and K^+^ the lowest for all steps (Q0→Q1: 159, 144, 136 kJ/mol; Q1→Q2: 90, 90, 62 kJ/mol; Q2→Q3: 166, 133, 92 kJ/mol; Q3→Q4: 131, 133, 121 kJ/mol, Li^+^ to K^+^, respectively) (see Figure 6c). Proton‐transfer barriers associated with TSHS_1 and TSHS_2 steps remain comparatively low and nearly cation‐independent. The overall decrease in activation energy from Li^+^ to K^+^ reflects the progressive reduction in positive charge density, which weakens cation‐oxygen electrostatic interactions and enhances the reactivity of negatively charged framework oxygens toward coordination with the Si monomer. This effect lowers the energetic cost of pentacoordinated transition states and favors defect healing in K‐containing systems. Consistent with this interpretation, transition‐state geometries show systematically increasing M–O distances from Li^+^ to K^+^, in line with larger ionic radii and reduced charge density (see Tables S4–S9). Reversing the Q0→Q4 pathway provides a mechanistic description of defect generation. Along this reversed Q4→Q0 route, all steps exhibit lower barriers and most intermediates are more stable in the presence of K^+^ compared to Na^+^ and Li^+^, indicating that potassium also favors defect formation (see Figure 6d). In line with this trend, Liu et al. [38, 39] reported a similar pathway for water‐mediated defect generation in MFI, with an activation energy of ∼200 kJ/mol, far exceeding the barriers identified in this work and underscoring the catalytic role of alkali cations in promoting annealing relative to water‐only mechanisms. While the precise origin of these defects, whether from pristine channels, Q4, or from pre‐existing Q3 species such as silanol nests and partially hydroxylated extra‐framework species, cannot be uniquely identified, the energy profile in Figure 6d consistently identifies the first hydrolysis step, Q4→Q3, as rate‐limiting for all three alkali cations. This observation points to pre‐existing Q3 configurations as especially favorable starting points for Si(OH)_4_ formation and vacancy‐site creation.

Free energy calculations using a self‐improving iterative umbrella sampling approach driven by a neural network based interatomic potential for zeolites were performed to evaluate Si(OH)_4_ diffusion in defective MFI containing a single silanol‐nest defect without water and alkali cations (see Figures S10–S12) [40, 41]. Helmholtz free‐energy profiles at 300 K for diffusion along both straight and sinusoidal channels indicate facile transport, with effective barriers  ≤30 kJ/mol. These results support the plausibility of hydrolysis and defect healing at room temperature, demonstrating that silica species present within the pores can reach vacancy sites.

Overall, both forward (healing) and reverse (cleavage) pathways demonstrate the indispensable roles of water and alkali cations. Water acts as an essential proton shuttle in key transition states (TSHS_1, TSHS_2 and TSQ3_Q4) and modulates cation charge density through coordination, enhancing framework oxygen reactivity. In contrast to water, whose role is primarily limited to proton shuttling and charge redistribution, the presence of an inorganic alkali cation is structurally indispensable to the cooperative annealing mechanism. Introduction of the cation necessarily occurs together with charge compensation by an associated hydroxyl anion, which preferentially binds axially to a framework silicon center along the zeolite channel, generating a pentacoordinated Si complex. This motif activates the framework toward Si–O bond cleavage and defect formation. Conversely, during defect healing, coordination of the alkali cation to the framework silanol nest creates a highly reactive environment that facilitates condensation upon approach of a Si(OH)_4_ monomer. In both cleavage and healing pathways, the alkali cation is therefore essential not merely for electrostatic stabilization, but for promoting the formation of negatively charged, pentacoordinated silicon intermediates that drive framework rearrangement.

### 2.3 Implications for Adsorption and Catalytic Applications

#### 2.3.1 Defect‐Free Silicalite‐1 as Potential Material for the Selective Adsorption of 1‐Butanol From Alcohol/Water Mixtures

Adsorption‐based processes have recently been identified as effective separation methods for the selective recovery of alcohols produced during biomass fermentations, such as 1‐butanol, from aqueous mixtures [9]. Among the various adsorbent candidates, hydrophobic zeolites have emerged as promising materials for the selective separation of alcohols from highly diluted aqueous solutions [42]. Achieving high butanol‐over‐water selectivity requires minimizing the presence of hydrophilic adsorption sites. In this context, the defect‐free Silicalite‐1 material described herein, which incorporates sub‐stoichiometric K content in the synthesis, exhibits ideal characteristics for addressing this demanding separation challenge.

The adsorption isotherms of water and 1‐butanol at 25°C for the Silicalite‐1 zeolites synthesized in the absence of alkali cations (S1_cal) and with sub‐stoichiometric K content (S1‐K_cal) are shown in Figure 3d. Notably, the S1‐K sample, which exhibits a lower concentration of structural defects, displays a significantly reduced water uptake compared with the defective S1 material at equivalent partial pressures (see filled red and green triangles in Figure 3d), consistent with its enhanced hydrophobicity. Both Silicalite‐1 samples show substantially higher 1‐butanol adsorption relative to water, and the 1‐butanol uptake is similar for both materials irrespective of their defect densities (see empty red and green rhomboids in Figure 3d). From the single‐component isotherms, the ideal 1‐butanol/water selectivity was calculated as a function of relative pressure (see Figure 3e). At P/P_0_ ∼0.1, the hydrophobic S1‐K material exhibits a selectivity at least five times higher than that of the hydrophilic S1 sample (∼18.5 and ∼3.5 for S1‐K_cal and S1_cal, respectively, see Figure 3e). In addition, the application of ideal adsorbed solution theory (IAST) to predict the binary 1‐butanol/water adsorption selectivity of samples S1 and S1‐K at 25°C has been included and discussed in the Supporting Information (see Figures S13–S15).

#### 2.3.2 Extension to Defect‐free Ti‐containing Silicalite‐1 (TS‐1) Materials: Synthesis, Characterization and Catalytic Properties for the Selective Epoxidation of Olefins

The superb and simple defect annealing process observed when calcining the pure siliceous Silicalite‐1 materials containing ultra‐low K contents trapped within the as‐prepared crystals opens unique opportunities to design industrially‐relevant heteroatom‐substituted zeolites, such as for instance, Ti‐containing Silicalite‐1 (TS‐1), minimizing the presence of structural defects in their frameworks without requiring the use of hazardous F sources. Having that in mind, we carried out the synthesis of the TS‐1 material under classical conditions by setting a Si/Ti molar ratio in the synthesis gel of ∼100 in the absence of K (TS1 material) and with the presence of a sub‐stoichiometric K content in the synthesis gel (TS1‐K material) (see Experimental Section for details).

After hydrothermal crystallization at 175°C for 3 days, the MFI structure was obtained in both cases (see Figure 7a), with similar crystal sizes (200–300 nm, see Figure 7b), BET surface areas and micropore volumes (∼373–390 m^2^/g and ∼0.17 cm^3^/g, see Table S1), but different N_2_ adsorption–desorption behaviors (see Figure 7c), consistent with distinct degrees of defect formation upon calcination inferred by ^2^ ^9^Si MAS NMR (see Figure 7d). Altogether, these results suggest the formation of a healed structure when K is present, as we had previously observed in Ti‐free analogues.

**FIGURE 7:**
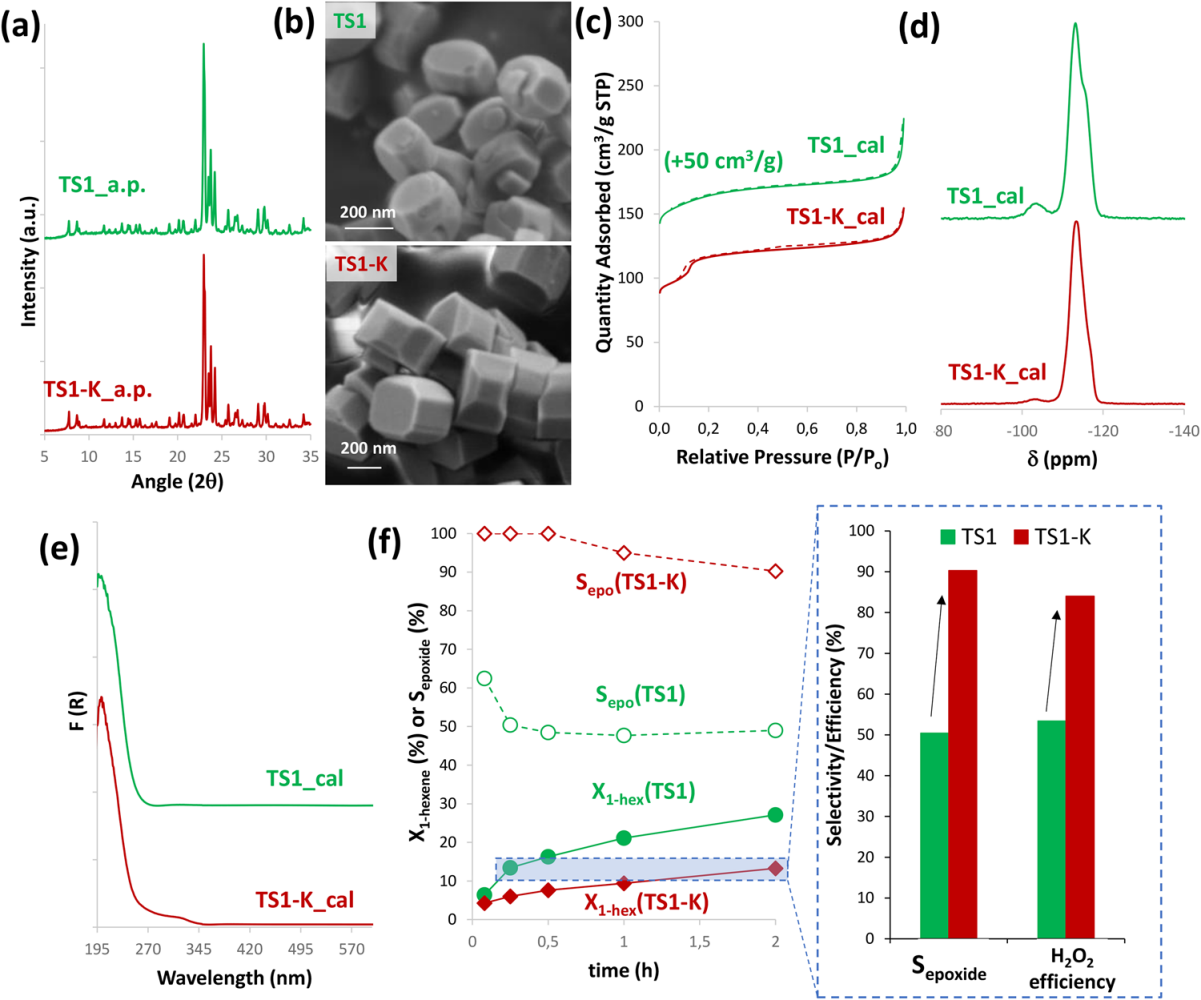
Characterization and catalytic activity of the Ti‐Silicalite‐1 materials prepared in absence (TS1) or presence of sub‐stoichiometric content of K cations (TS1‐K). (a,b) PXRD patterns (a) and FESEM images (b) of the as‐prepared TS‐1 materials. (c–e) N_2_ adsorption/desorption isotherms at 77 K (c), ^29^Si MAS NMR spectra (d) and UV–vis spectra (e) of the calcined TS‐1 materials. (f) 1‐hexene conversion, 1,2‐epoxyhexane selectivity and H_2_O_2_ efficiency for the 1‐hexene epoxidation reaction with H_2_O_2_ as oxidant in methanol at 60°C using the calcined TS‐1 materials as catalysts (Reaction conditions: 15 mg of catalyst, 1.4 mmol of 1‐hexene, 1.4 mmol of H_2_O_2_, 1.4 mL of methanol, 60°C). *Note*: The characterization and catalytic results presented here for the calcined TS1‐K material have been acquired after removing the inorganic cations.

The chemical analysis of TS1 and TS1‐K reveals a similar Ti content in the final materials, with a Si/Ti molar ratio of ∼80‐87 (see TS1 and TS1‐K in Table 1) and, interestingly, the UV–vis spectra of the calcined TS1 and TS1‐K samples clearly reveal the almost entire presence of the Ti species in tetrahedral coordination in framework positions of the MFI structure as indicated by the intense band at 210–220 nm (see Figure 7e). These species have been extensively assigned in the literature as the active sites to undergo the selective epoxidation of olefins, such as 1‐hexene, using H_2_O_2_ as oxidant agent. Therefore, two TS‐1 materials have been synthesized with analogous crystal size, chemical composition, and crystallinity, but different content of defective sites in their structure, resulting in different hydrophobic/hydrophilic properties that could seriously influence the catalytic performance when studying selective oxidation reactions of olefins with the aim of maximizing epoxide products.

Consequently, the calcined TS1 and TS1‐K materials have been tested as catalysts for the selective oxidation of 1‐hexene using H_2_O_2_ as oxidant and methanol as solvent at 60°C (see Experimental Section for details). The more hydrophilic TS1 material, which presents a significantly higher number of defects, shows an enhanced 1‐hexene conversion compared to the more hydrophobic TS1‐K catalyst (see filled green circles and filled red rhombs for 1‐hexene conversion values with time for TS1 and TS1‐K catalysts in Figure 7f). However, the selectivity toward the desired 1,2‐epoxyhexane product is noticeably lower when using the hydrophilic TS1 catalyst (below 60%, see empty green circles in Figure 7f) compared to the more hydrophobic TS1‐K catalyst (above 90%, see empty red rhombs in Figure 7f), regardless of the 1‐hexene conversion values. In fact, if the catalytic behavior of both TS‐1 catalysts is compared at the same conversion level (13%–14%, see Figure 7f‐inset), not only the selectivity toward the desired 1,2‐epoxyhexane product is substantially higher with the hydrophobic TS1‐K catalyst compared to the alkali‐free hydrophilic TS1 catalyst (90.3 and 50.4 with TS1‐K and TS1, respectively, see Figure 7f‐inset), but also the efficiency of H_2_O_2_ utilization in the 1‐hexene epoxidation reaction is vastly higher with the hydrophobic TS1‐K catalyst (84.0 versus 53.4 with TS1‐K and TS1, respectively, see Figure 7f‐inset). These results clearly reflect the importance of designing hydrophobic catalysts by using simple synthetic strategies as the one presented here by introducing sub‐stoichiometric K contents in the synthesis preparations of the MFI‐type materials. This simple post‐synthetic approach could be easily extended to other zeolite structures with different chemical compositions and, consequently, with tremendous implications in challenging catalytic processes, such as, for instance biomass‐derived processes.

## 3 Conclusions

This work demonstrates that sub‐stoichiometric amounts of alkali cations (Na^+^ and K^+^) in the fluoride‐free synthesis of Silicalite‐1 play a key role in controlling framework defects and tuning textural and surface properties after calcination. DFT simulations and high‐resolution microscopy support a defect‐healing mechanism in which in‐situ generated NaOH or KOH species promote Si–O–Si bond rearrangement and facilitate the migration of Si(OH)_4_ species, enabling the effective healing of T‐site vacancies and leading to highly ordered, defect‐free MFI structures. The defect‐free Silicalite‐1 exhibited enhanced selectivity in butanol/water separation, highlighting the importance of defect control in tuning hydrophobicity and adsorption properties. These insights were successfully extended to the synthesis of defect‐free TS‐1 materials, resulting in hydrophobic Lewis acid catalysts with improved performance in the selective epoxidation of 1‐hexene. Overall, this simple and scalable strategy provides an effective route to obtain defect‐free zeolites with tailored physicochemical properties in the absence of hazardous fluoride, opening new opportunities for the design of advanced catalysts and separation materials, particularly for processes involving water or polar compounds, such as biomass‐derived transformations. This work also highlights that even trace amounts of cations not intentionally included in the synthesis can dramatically affect the structural properties of the resulting zeolite, leading to poor reproducibility or erroneous conclusions if not properly accounted for. This emphasizes the broader relevance of our findings for the rational design and interpretation of zeolite syntheses.

## 4 Experimental Section/Methods

### 4.1 Zeolite Synthesis

#### 4.1.1 Materials

The following materials have been used in this study: Tetraethyl orthosilicate (TEOS, Sigma‐Aldrich) as silicon source, tetrapropylammonium hydroxide [TPAOH (SA): Sigma Aldrich, 20 wt% aqueous solution, 0.5 wt% K, LOT: 102749509, 102633249; TPAOH (TS), Thermo Scientific, 40 wt% aqueous solution, LOT: P221062] as template agent, aluminum hydroxide (Thermo Scientific, 76.5 wt%), potassium chloride (KCl, Sigma Aldrich), sodium chloride (NaCl, Sigma Aldrich), titanium (IV) butoxide (TBOT, Sigma‐Aldrich, reagent grade, 97%), 2‐propanol (IPA, Sigma‐Aldrich) and MilliQ water.

#### 4.1.2 Synthesis of Silicalite‐1 Materials

The hydrothermal synthesis of Silicalite‐1 with Na (S1‐Na) was as follows: 48 mg of NaCl was mixed with 2.85 g of TPAOH (TS) and 6.13 g of water. When NaCl is dissolved, 4.17 g of TEOS was added, and the mixture stirred overnight until the hydrolysis of TEOS in a closed vessel. The composition of the mixture was: 1 SiO_2_: 0.28 TPAOH: 0.036 NaCl: 40 H_2_O: 4 EtOH. The mixture was transferred into a sealed Teflon‐lined autoclave and placed in an oven at 175°C for 3 days. After crystallization, the solid product was obtained by centrifugation at 6000 rpm, washed with ethanol and water, dried overnight at 80°C, and calcined in air at 580°C for 4 h.

The procedure was repeated without the addition of NaCl to create a composition of 1 SiO_2_: 0.28 TPAOH: 40 H_2_O: 4 EtOH for the alkali‐free Silicalite‐1 (S1).

Finally, a third synthesis mixture was prepared similarly without the addition of NaCl and substituting the use of TPAOH (TS) by adding 5.61 g of TPAOH (SA), achieving the following mixture composition for the Silicalite‐1 with K (S1‐K): 1 SiO_2_: 0.28 TPAOH: 0.036 K: 40 H_2_O: 4 EtOH. It is worth noting that the addition of the same amount of K^+^ in the form of KCl, using ultra‐high purity TPAOH (TS), allows obtaining a Silicalite‐1 sample with analogous properties.

After the calcination, the alkali cations were substituted with ammonium (NH_4_^+^) during an ion exchange. The material was treated with ammonium chloride (NH_4_Cl, 1 M) in a liquid/solid ratio = 10. The exchange took place in a silicone oil bath at 80°C for 2 h with vigorous stirring. Afterwards, the material was washed with distilled water and calcined in a fixed‐bed reactor with air at 550°C for 4 h with a heating ramp of 2°C/min.

Three additional experiments (S1‐K_0.01_, S1‐K_0.006_, S1‐K_0.003_) were performed by fixing the K/Si ratios in the synthesis mixtures at 0.01, 0.006, and 0.003, respectively, in order to determine the minimum K content required to efficiently perform the defect annealing in Silicalite‐1. This was achieved using 2.85 g of ultra‐high‐purity TPAOH (TS) and 3.63, 2.18, or 1.09 g of a KCl solution (0.50 wt% K) as the potassium source, respectively, achieving the following mixture compositions: 1 SiO_2_: 0.28 TPAOH: 0.01‐0.006‐0.003 KCl: 40 H_2_O: 4 EtOH. The ^29^Si MAS NMR spectra of the crystallized samples, after appropriate calcination and cation exchange treatments, are shown in Figure S16. The sample prepared with a K^+^/Si ratio of 0.01 exhibits clear defect annealing (see S1‐KCl_0.01_Cal_ in Figure S16). When the K^+^ content is further reduced to 0.006 and 0.003, partial defect healing is still observed; however, in these cases, the efficiency of the process decreases, particularly when potassium content becomes too low (see S1‐KCl_0.003_Cal_ in Figure S16). These preliminary results suggest the existence of an optimum potassium content for MFI‐type materials, which is necessary to effectively promote defect healing and obtain truly hydrophobic zeolite frameworks.

#### 4.1.3 Synthesis of Ti‐Silicalite‐1 Materials

The hydrothermal synthesis of Ti‐containing Silicalite‐1 (TS1) was as follows: 3.80 g of TPAOH (TS) was mixed with 9.72 g of water. Then, 6.21 g of TEOS was added, and the mixture was stirred for 2 h until the hydrolysis of TEOS (solution 1). In parallel, a second solution was prepared, made of 0.86 g of TBOT and 4.99 g of IPA (solution 2). After 2 h, 0.78 g of solution 2 was added to solution 1 and the final gel was maintained under stirring overnight. The composition of the mixture was: 1 SiO_2_: 0.01 TiO_2_: 0.25 TPAOH: 35 H_2_O: 0.33 IPA: 4 EtOH. The mixture was transferred into a sealed Teflon‐lined autoclave and placed in an oven at 175°C for 3 days. After crystallization, the solid product was obtained by centrifugation at 6000 rpm, washed with ethanol and water, dried overnight at 80°C, and calcined in air at 580°C for 4 h.

The procedure was repeated with the addition of 3.63 g of a KCl solution (0.50 wt% of K) to the solution 1 simultaneously with TPAOH (TS) and water, in order to synthesize TS‐1 containing K (TS1‐K), resulting in the following molar composition: 1 SiO_2_: 0.01 TiO_2_: 0.25 TPAOH: 0.01 KCl: 35 H_2_O: 0.33 IPA: 4 EtOH.

After the calcination, the alkali cations were substituted with ammonium (NH_4_^+^) during an ion exchange. The material was treated with ammonium chloride (NH_4_Cl, 1 M) in a liquid/solid ratio = 10. The exchange took place in a silicone oil bath at 80°C for 2 h with vigorous stirring. Afterwards, the material was washed with distilled water and calcined in a fixed‐bed reactor with air at 550°C for 4 h with a ramp of 2°C/min.

### 4.2 Characterization

Powder x‐Ray diffraction (PXRD) measurements were performed employing a multisample holder Panalytical X'Pert diffractometer, running at 35 mA and 40 kV and a Cu Kα radiation of 0.1542 nm of wavelength.

The chemical composition of the materials was determined by inductively coupled plasma optimal emission spectroscopy (ICP‐OES) using a Thermo‐Scientific iCAP PRO spectrometer after dissolving the solid samples in an acid mixture of HNO_3_: HCl: HF (1:3:1 volumetric ratio).

The morphologies and crystal sizes of the different zeolite particles were studied by high resolution field emission scanning electron microscopy (HR‐FESEM) employing a ZEISS GeminiSEM 500 microscope (ZEISS‐ Oxford Instruments) fitted with a Backscattered detector.

Textural properties were determined by N_2_ adsorption isotherms measured at 77 K with a Micromeritics ASAP 2020. Specific surface areas, using the Brunauer–Emmett–Teller model, were extrapolated, fulfilling the criterion established by Rouquerol and pore volume was calculated by the t‐plot method [43].

Solid state MAS NMR spectra were recorded with a Bruker AV400 III HD spectrometer. Prior to recording the ^1^H MAS NMR spectra, the samples were evacuated in vacuum at 150°C to remove the adsorbed water on the zeolite surface. The spectra were recorded using a Bruker 3.2 mm probe by spinning the sample at 20 kHz, and a recycle delay of 15 s. ^29^Si MAS NMR spectra were recorded using a Bruker 7 mm probe at a spinning rate of 5 kHz with a π/3 pulse length of 3.5 µs and a repetition time of 180 s. ^1^H and ^29^Si chemical shifts were referenced to tetramethylsilane.

The UV−vis spectra were collected using an Agilent Cary 7000 UV−vis−NIR spectrophotometer equipped with a diffuse reflectance accessory.

The Fourier transformed infrared spectra were collected in a Nicolet 710 FTIR device. Self‐supported wafers (10 mg/cm^2^) of the samples were previously degasified overnight at 400°C and dynamic vacuum (10^−6^ mbar). After activation, the base spectrum was recorded at 150°C. All the spectra were normalized to the area of the corresponding Si‐O overtones (1755–2100 cm^−1^).

Transmission electron microscopy (TEM) was carried out in scanning mode (S)TEM using an annular dark field detector (ADF) for data collection. The equipment used was a Thermo Fisher Titan low base operated at 300 kV. The column was fitted CEOS spherical aberration corrector (Cs‐corrected). Aberrations were minimized using a gold standard sample prior to every measurement assuring a spatial resolution of 0.8 Å. For analytical measurements, the TEM was equipped with an EDS (energy dispersive x‐ray spectroscopy) Oxford Instruments Ultim Max TLE 100.

### 4.3 Theoretical Calculations and Models

Periodic DFT calculations were performed using the Perdew–Burke–Ernzerhof (PBE) exchange–correlation functional within the generalized gradient approximation (GGA) [44], employing the Vienna ab initio simulation package (VASP) [45]. The valence electronic states were represented with a plane‐wave basis set using a kinetic‐energy cutoff of 600 eV, while interactions between core and valence electrons were described using the projector augmented‐wave (PAW) approach [46]. Sampling of the Brillouin zone was limited to the Γ‐point. Long‐range dispersion was incorporated through Grimme's D3 correction [47, 48]. Electronic self‐consistency was achieved with a convergence threshold of 10^−^^6^ eV, and geometry optimizations were carried out until all atomic forces were below 0.01 eV/Å. Transition‐state structures were identified using the DIMER algorithm [49, 50]. All atoms were allowed to relax freely during the optimization steps. The zeolite‐confined transition states obtained in this manner were subsequently examined by harmonic frequency analysis to confirm the presence of a single imaginary mode and to rule out higher‐order saddle points.

To model the MFI structure, we used a unit cell with 288 atoms (96 T and 192 O) with lattice parameters a = 20.010 Å, b = 19.943 Å, c = 13.326 Å, α = 90.00°, β = 90.00°, γ = 90.00° obtained after full relaxation of the experimental lattice parameters. The defective site was created by removing a Si atom from a T3 site, in agreement with previous ^29^Si MAS NMR and PXRD studies [37]. The removed Si atom was left as a Si(OH)_4_ unit within the channel, while the resulting vacancy was stabilized by a silanol nest consisting of three OH groups and one OM (M = K, Na, Li) group, and a coordinated water molecule.

To investigate Si(OH)_4_ diffusion, a Si vacancy was generated by removing a Si atom from the T3 site at the channels intersection. The vacancy was stabilized by a neutral silanol nest consisting of four OH groups, and the removed Si atom was left as the Si(OH)_4_ unit that diffuses through the MFI channels system. For these calculations, a machine learning interatomic potential (MLIP) was employed, trained using the PaiNN architecture [51], on the same dataset described in a previous work [41] at the r^2^SCAN+D4 meta‐GGA DFT level of theory, employing the SchNetPack 2.0 Python package [52]. The MLIPs used three interaction blocks, a feature vector size of 128, and utilized 20 Bessel radial basis functions for the expansion of pairwise distances with a cutoff radius of 5 Å. A trainable ZBL short‐range repulsion term was added to the total energy expression. The resulting NNPs showed energy and force root mean square errors of 1.6 meV per atom and 63.3 meV Å^−1^ for the database test set, respectively.

Molecular dynamics simulations employing the MLIP were carried out within the atomistic simulation environment (ASE) [53] in the NVT ensemble, using a Langevin thermostat with a friction coefficient of 0.01 at 300 K with a timestep of 0.5 fs. Harmonic restraints were applied to the molecular dynamics simulations with the PLUMED library in the version 2.9.2 [54]. 15 artificial harmonic umbrella potentials acting on the center of mass (COM) of the Si(OH)_4_ molecule were applied to the model, with minima evenly distributed along the CV space. Biased models were optimized using the BFGS algorithm in ASE, with the maximum distance that an atom could move per iteration set to 0.01 Å. The convergence criterion was that the force acting on each atom should be less than 0.01 eV/Å. The Si(OH)_4_ molecule was moved from the initial position of the COM to the respective position of the umbrella window during optimization. Subsequently, each simulation was equilibrated for 0.5 ps, after which followed a production run for 50 ps for each window. Umbrella sampling data were decorrelated and analyzed using the timeseries module and the multistate Bennet‐acceptance‐ratio (MBAR) method in the Pymbar Python package [55, 56]. Force constants and window spacing were iteratively optimized using the optimal grid refinement (OGRe) algorithm [57]. The accuracy of the FES was assessed using an automated in‐house implementation of the OGRe protocol, which evaluates: (i) overlap between neighboring windows, (ii) confinement within window boundaries, and (iii) consistency with the reweighted distribution. Initial force constants in umbrella windows were set as 300 kJ/(mol Å).

### 4.4 Adsorption and Catalytic Tests

#### 4.4.1 Adsorption of Water and 1‐Butanol

Highly pure 1‐butanol (1‐BuOH, Merck Life Science S.L.U, 99.9%) and MilliQ water were used in the adsorption experiments. The adsorbates were subjected to freeze‐pump‐thaw cycles before being used to ensure the absence of gaseous impurities.

A BelSorp Max II volumetric device was used for measuring high resolution isotherms of 1‐butanol and water vapors at 25°C. Highly pure helium (Air Liquide, 99.996%) was used for the dead volume determination.

In all cases, activation of the samples at 350°C under high turbomolecular vacuum was carried out prior to the measurement.

The ideal adsorbed phase selectivity for 1‐butanol (1‐BuOH) relative to water can be estimated from their pure‐component adsorption isotherms under identical partial pressure. It is expressed as the ratio of the equilibrium uptake of 1‐butanol to that of water at the same partial pressure and temperature. This metric is given by S_1‐BuOH/H2O_ = n_1‐BuOH_/n_H2O_, and provides a first approximation of the preferential adsorption of the alcohol over water on the selected adsorbent. Although this approach neglects competitive adsorption and non‐ideal interactions present in real mixtures, it is widely used for preliminary screening of adsorbents in separation processes and will clearly point out the preferred selectivity of one adsorbate over the other. Comparing these values across different materials highlights the influence of surface polarity, allowing a clear distinction between hydrophilic and hydrophobic adsorbents in terms of their affinity for alcohol versus water.

#### 4.4.2 Epoxidation of 1‐Hexene

To evaluate the catalytic activity of the samples, the liquid‐phase oxidation of 1‐hexene (Sigma‐Aldrich, 97%) with hydrogen peroxide (H_2_O_2_) (Fisher Scientific, Honeywell Fluka, 30% in aqueous solution) was carried out in a 3 mL glass reactor. In a typical reaction, 15 mg of catalyst, 1.4 mL of methanol, 1.4 mmol of 1‐hexene, and 1.4 mmol of H_2_O_2_ were introduced into a glass reactor and heated at 60°C under vigorous magnetic stirring (800–1000 rpm) for the desired reaction time. After completion of the reaction, the reactor was immediately immersed in an ice bath and cooled until the internal temperature reached <5°C (approximately 5 min). Next, 0.15 mL of the internal standard, cyclohexanone (>99%, Sigma‐Aldrich), was added. Finally, the mixture composed of the reaction crude and cyclohexanone was homogenized with 1.5 mL of isopropanol (HPLC‐grade, Scharlau). The reaction evolution was analyzed in a Shimadzu Nexis 2030 using an HP‐5 column (30 x 0.25 x 0.25).

In addition, the remaining hydrogen peroxide (H_2_O_2_) was quantified by cerimetric titration using 0.1 M Ce(SO_4_)_2_ as oxidant in sulfuric acid medium. The Ce(IV) titrant was prepared by dissolving high‐purity cerium(IV) sulfate (≥99%, Sigma‐Aldrich) in 0.92 M H_2_SO_4_ to obtain a nominal concentration of 0.1 M. The solution was freshly prepared and protected from light prior to use. The ferroin indicator solution was prepared by dissolving 0.174 g of ferrous sulfate (FeSO_4_·7H_2_O, >99%, Sigma‐Aldrich) in 25 mL of deionized water, followed by addition of 0.371 g of 1,10‐phenanthroline monohydrate (99%, Sigma‐Aldrich). The mixture was stirred until complete dissolution, affording a deep red solution, which was stored protected from light.

In a typical titration, the reaction mixture was filtered to remove the solid catalyst, and the clear filtrate was transferred into a 250 mL Erlenmeyer flask containing 150 mL of 0.92 M H_2_SO_4_ pre‐cooled to 5°C to minimize non‐catalytic decomposition of H_2_O_2_. Subsequently, 2‐3 drops of the ferroin indicator solution were added, and the solution was titrated under continuous stirring with the prepared 0.1 M Ce(SO_4_)_2_ until the appearance of a stable pale blue coloration persisting for at least 5 s, indicating a slight excess of Ce^4^^+^.

Control titrations of the initial H_2_O_2_ stock solution were carried out under identical conditions to verify the accuracy and reproducibility of the method. The H_2_O_2_ concentration was calculated considering that two equivalents of Ce^4^^+^ react per mole of H_2_O_2_.

## Conflicts of Interest

The authors declare no conflicts of interest.

## Supporting information




**Supporting File**: adma74455‐sup‐0001‐SuppMat.docx.

## Data Availability

The data that support the findings of this study are available from the corresponding author upon reasonable request.
