# Supplementary material for: Hydrophobic MFI‐Type Zeolites via Alkali‐Cation‐Induced Defect Healing: Implications for Adsorbent and Catalyst Design
Source: Adv Mater. 2026 Aug 5;38(56):e74455. doi: 10.1002/adma.74455 (PMC13648907; doi:10.1002/adma.74455)
Supplement: Supplementary file 1 [file ADMA-38-e74455-s001.docx]

Hydrophobic MFI-Type Zeolites via Alkali-Cation-Induced Defect Healing: Implications for Adsorbent and Catalyst Design

*Christos Kanteler,^1+^ Pau Ferri,^1+^ Francisco Javier Escobar-Bedia,^1^ Álvaro Mayoral,^2^ Alejandro Vidal-Moya,^1^ Richard Kendra,^3^ Chen Lei, ^3^ Miguel Palomino,^1^ Fernando Rey,^1^ Lukáš Grajciar,^3^ Christopher James Heard,^3^ Pedro Serna,^1^ Mercedes Boronat,^1^* Cristina Martínez,**^1^* Manuel Moliner^1^**

^1^ Instituto de Tecnología Química, Consejo Superior de Investigaciones Científicas-Universitat Politècnica de València, 46022 València, Spain
E-mail: boronat@itq.upv.es; cmsanche@itq.upv.es; mmoliner@itq.upv.es

^2^ Instituto de Nanociencia y Materiales de Aragón (INMA), CSIC-Universidad de Zaragoza, 50009 Zaragoza, Spain
^3^ Department of Physical and Macromolecular Chemistry, Charles University, 12843 Prague, Czech Republic

*** Corresponding authors: boronat@itq.upv.es; cmsanche@itq.upv.es; mmoliner@itq.upv.es

+ These authors contributed equally

**Supporting Information**

**Figure S1.** (a) Nest that results from an absent T site to produce three surrounding silanols (Si–OH) and one siloxy (Si–O^-^). (b) Nest produced by breaking at least one siloxane (Si–O–Si) linkage to create one siloxy and one silanol. M^+^ can be TPA^+^ or K^+^ in the as-prepared K-MFI system. Reproduced from ref.^[1]^

**Figure S2.** Thermogravimetric analyses (TGA) of the different Silicalite-1 (a) and Ti-Silicalite-1 materials in their as-prepared forms prepared in absence or presence of sub-stoichiometric content of K cations.

**Figure S3.** (a) Low magnification Cs-corrected STEM-ADF of different particles of S1-K_a.p. (b) Spectrum profile obtained from the analysis of Figure 2.

**Figure S4.** Schematic representation along a) sinusoidal b) straight MFI channel of **TSQ0_Q1** showing atom labeling used for the distance measurements reported in Table S4. Oxygen, silicon, hydrogen, and alkali cations (M⁺) are shown in red, yellow, white, and purple, respectively. Oxygen labels O1–O4, consistent across Figures S5-S9, correspond to the four oxygen atoms that ultimately bond to the Si(OH)₄ monomer during vacancy sealing. Labels O5–O8 indicate the oxygens of the OH groups that are removed as water during the process. In each transition state, the oxygen with the highest index is the one eliminated from the Si(OH)₄ monomer: O8 in TSQ0_Q1, O7 in TSQ1_Q2 (Figure S6), O6 in TSQ2_3 (Figure S8) and O5 in TSQ3_Q4 (Figure S9). Label O9 corresponds to the water molecule already present in the Q0 model. In TSQ0_Q1, TSQ1_Q2, and TSQ2_Q3, H1 labels the proton transferred from O5 to the leaving oxygen to form a water molecule, while H2 corresponds to the proton of the departing OH group. For the specific cases TSHS_1, TSHS_2, and TSQ3_Q4, the labeling scheme is described in the corresponding captions of Figures S5, S7, and S9. M^+^ label accounts for the different alkali cations studied; Li^+^, Na^+^ and K^+^.

**Figure S5.** Schematic representation along a) sinusoidal b) straight MFI channel of TSHS_1 showing the atom labeling used for the distance measurements reported in Table S5. The atom labeling scheme is described in the caption of Figure S4**.** H1 labels the proton transferred from O2 to the O8 water molecule, and H2 labels the proton transferred from the water molecule to O5.

**Figure S6.** Schematic representation along a) sinusoidal b) straight MFI channel of **TSQ1_Q2** showing atom labeling used for the distance measurements reported in Table S6. The atom labeling is described in the caption of Figure S4.

**Figure S7.** Schematic representation along a) sinusoidal b) straight MFI channel of **TSHS_2** showing atom labeling used for the distance measurements reported in Table S7. The atom labeling scheme is described in the caption of Figure S4. H1 labels the proton transferred from O3 to the O7 water molecule, and H2 labels the proton transferred from the water molecule to O5.

**a)	b)**

**Figure S8.** Schematic representation along a) sinusoidal b) straight MFI channel of **TSQ2_Q3** showing atom labeling used for the distance measurements reported in Table S8. The atom labeling is described in the caption of Figure S4.

**Figure S9.** Schematic representation along a) sinusoidal b) straight MFI channel of **TSQ3_Q4** showing atom labeling used for the distance measurements reported in Table S8. The atom labeling is described in the caption of Figure S4. H1 labels the proton transferred from O4 to the O6 water molecule, and H2 labels the proton transferred from the water molecule to O5.

**a)	b)**

**Figure S10.** Helmholtz free energy profiles for the diffusion of Si(OH)_4_ through the channel system of siliceous MFI containing a silanol nest defect at the channels intersection, as calculated from umbrella sampling simulations at 300 K. (a) Sinusoidal channel diffusion: Si(OH)₄ near the channel intersection interacting with the defect (a1); transferred into the straight channel while maintaining defect interaction (a2); centered in the sinusoidal channel without significant interactions with the channel walls (a3); at the center of a 10-membered ring, which is the most constrained point of the diffusion process, and corresponds to the highest free energy barrier (a4); in a dispersive pocket after passing through the 10-ring (a5); at the transition region at the center of the next 10-membered ring along the diffusion path (a6). (b) Straight channel: at the intersection approaching the straight-channel 10-ring (b1); in the sinusoidal channel interacting with the defect (b2); closer to the channel intersection (b3); in the straight channel near the intersection interacting with the defect (b4) (global minimum, corresponding to a2); moving away from the silanol nest along the straight channel (b5); in a dispersive pocket beyond the 10-ring (b6); transition state for passage through the straight-channel 10-membered ring (b7).

**Figure S11.** (a) Top view showing Si(OH)₄ protruding through the 10-membered ring of the sinusoidal channel, corresponding to the highest diffusion barrier. The channel outline is highlighted with a dashed line. (b) View along the sinusoidal channel. (c) Top view showing Si(OH)₄ in a dispersive pocket within the sinusoidal channel after passing through the 10-membered ring (local minimum).

**Figure S12.** (a) Cutaway top view showing the interaction of Si(OH)₄ with the silanol-nest defect inside the straight channel. (b) View along the straight channel highlighting the interaction between Si(OH)₄ and the defect. (c) View along the straight channel with Si(OH)₄ located at the center of the pore. (d) Side view showing Si(OH)₄ far from the defect and partially protruding through the highlighted 10-membered ring, corresponding to the highest diffusion barrier.

**Figure S13.** Experimental isotherms of butanol and water on Si and S1-K and the fittings using a number of different adsorption parametric models and PCHIP interpolation curves.

Ideal Adsorbed Solution Theory (IAST) has been applied to predict the binary 1-butanol/water adsorption selectivity of samples S1 and S1-K at 298 K.

Pure-component adsorption isotherms of water and 1-butanol were measured for both materials at 298 K. A qualitative inspection of the isotherms already indicates that 1-butanol is preferentially adsorbed over water on both samples. However, the water isotherm of S1 shows a non-negligible uptake at very low water pressures, suggesting that water adsorption may affect the 1-butanol/water selectivity under these conditions. To quantify this effect, IAST calculations were performed.

As a first step, the experimental isotherms were fitted using two complementary approaches: (i) the best-fitting parametric model selected from the 15-model library implemented in RUPTURA,^[2]^ and (ii) a shape-preserving Piecewise Cubic Hermite Interpolating Polynomial (PCHIP) interpolation,^[3,4]^ that passes exactly through the experimental data points of the isotherms.

The best-fitting models were a dual-site Langmuir (DSL) for water and a Toth model for 1-butanol on S1, and a Bingel-Walton model for water and a Toth model for 1-butanol on S1-K (all with the highest R2 coefficients in their respective rankings, being R2 = 0.9963, 0.9983, 0.9938 and 0.9978, respectively) as shown in Figure S13.

IAST calculations were carried out using both the parametric models and the PCHIP interpolations to determine the adsorbed-phase composition and the corresponding selectivity as a function of gas-phase composition. The calculations followed the implementation available in RUPTURA,^[2]^ which was also used to validate the parametric approach.

Both representations produced very similar results. The predicted selectivities were 20.2 and 18.9 for S1, and 91.8 and 95.2 for S1-K, using the parametric models and the PCHIP interpolation, respectively. The agreement, within approximately 4–6%, and the nearly overlapping selectivity curves demonstrate that the IAST predictions are robust and are not significantly affected by the choice of isotherm representation, particularly for the water isotherm, which is the most challenging to fit.

It should be noted that these values correspond to ideal IAST selectivities. Therefore, they do not account for non-ideal effects in the adsorbed phase, such as water clustering or asymmetric competition within the MFI pore network. Consequently, the calculated selectivities should be considered as ideal reference values, while their quantitative validation would require binary co-adsorption experiments.

**Figure S14.** Calculated IAST isotherms for 1-butanol and water as a function of gas-phase composition (1-butanol) at total pressures of 0.05, 0.4, and 0.8 kPa on samples S1 and S1-K using the best parametric model-fittings.

Figure S14 shows the predicted IAST 1-butanol/water selectivities as a function of gas-phase composition at total pressures of 0.05, 0.4, and 0.8 kPa. Both materials exhibit organophilic behaviour over most of the entire gas-phase composition range, with 1-butanol being preferentially adsorbed under almost all conditions. However, at very low 1-butanol partial pressures, S1 preferentially adsorbs water, whereas S1-K maintains a preference for 1-butanol. This result confirms the higher hydrophobicity of the K-containing sample.

**Figure S15.** Calculated butanol/water IAST selectivities on samples S1 and S1-K as a function of gas-phase composition (1-butanol) at total pressures of 0.05, 0.4, and 0.8 kPa.

The calculated selectivities further highlight the superior performance of S1-K (see Figure S15). At a total pressure of 0.8 kPa, S1-K exhibits a nearly constant 1-butanol/water selectivity of approximately 100 over the entire range of gas-phase compositions. In contrast, the selectivity of S1 increases from about 5 to 20 as the 1-butanol partial pressure increases. Thus, S1-K is consistently much more selective for 1-butanol adsorption than S1 under all investigated conditions.

Increasing the total pressure does not alter this trend. As shown in Figure S13, the selectivity of S1-K remains very high, or even increases slightly, indicating that its outstanding preference for 1-butanol over water is maintained over the investigated pressure range. These results suggest that the enhanced hydrophobicity of the K-containing zeolite is preserved under conditions relevant to practical separation processes.

**Figure S16.** ^29^Si MAS NMR spectra and of the S1-K samples prepared using KCl as potassium source with the following K/Si molar ratios in the synthesis mixture (0.003, 0.006 and 0.01) after calcination and cation exchange processes (Note: the ICP analyses correspond the S1-K samples in their as-prepared forms).

**Table S1.** Elemental analyses of the as-prepared Silicalite-1 materials prepared in absence or presence of sub-stoichiometric K content (S1_a.p. and S1-K_a.p., respectively).

| **Sample^a^** | **Si/M** | **Cat** | **N**  **(wt. %)** | **C**  **(wt. %)** | **C/N (mol/mol)** | **TPA/u.c.^b^** |
| --- | --- | --- | --- | --- | --- | --- |
|  | ***Si*** |  |  |  |  |  |
| S1_a.p. | ∞ | -- | 0.88 | 9.20 | 12.2 | 4.2 |
| S1-K_a.p. | ∞ | K | 0.89 | 9.19 | 12.1 | 4.1 |
|  |  |  |  |  |  |  |
|  |  |  |  |  |  |  |
|  |  |  |  |  |  |  |

**Table S2.** Electronic energies (kJ/mol) along (a) the healing mechanism Q0 → Q4 and (b) the cleaving mechanism Q4 → Q0 at a T3 framework vacancy in MFI, for each alkali cation (Li⁺, Na⁺, K⁺). The corresponding numerical values are plotted in Figure 6 of the main text. M_M and M_F denote configurations in which the alkali cation is coordinated either to an oxygen atom of the Si(OH)₄ monomer (M_M) or to a framework oxygen (M_F).

| a) **Defect Healing** | | | | b) **Defect Cleaving** | | | |
| --- | --- | --- | --- | --- | --- | --- | --- |
| **Step** | **Li⁺** | **Na⁺** | **K⁺** | **Step** | **Li⁺** | **Na⁺** | **K⁺** |
| Q0 | 0 | 0 | 0 | Q4 | 0 | 0 | 0 |
| TSQ0_Q1 | 159 | 144 | 136 | TSQ4_Q3 | 154 | 145 | 128 |
| Q1-M_M | 71 | 60 | 58 | Q3-M_M | 23 | 12 | 7 |
| TSHS_1 | 108 | 106 | 90 | TSQ2_Q3 | 138 | 97 | 83 |
| Q1-M_F | 23 | 18 | 15 | Q2-M_F | -28 | -35 | -8 |
| TSQ1_Q2 | 113 | 108 | 77 | TSHS_2 | 74 | 55 | 38 |
| Q2-M_M | 4 | -2 | -17 | Q2-M_M | -5 | -14 | -30 |
| TSHS_2 | 83 | 67 | 51 | TSQ1_Q2 | 105 | 95 | 64 |
| Q2-M_F | -19 | -23 | 4 | Q1-M_F | 14 | 6 | 3 |
| TSQ2_Q3 | 147 | 110 | 96 | TSHS_1 | 100 | 93 | 77 |
| Q3-M_M | 32 | 24 | 20 | Q1-M_M | 63 | 47 | 45 |
| TSQ4_Q3 | 162 | 158 | 141 | TSQ0_Q1 | 151 | 132 | 123 |
| Q4 | 9 | 13 | 13 | Q0 | -9 | -13 | -13 |

**Table S3.** Activation energy barriers (kJ/mol) for the a) healing and b) cleaving of a T3 framework vacancy through the annealing mechanism in MFI. Values are reported for Li⁺, Na⁺, and K⁺ cations.

| a) **Defect Healing** | | | | b) **Defect Cleaving** | | | |
| --- | --- | --- | --- | --- | --- | --- | --- |
| **Step** | **Li⁺** | **Na⁺** | **K⁺** | **Step** | **Li⁺** | **Na⁺** | **K⁺** |
| TSQ0_Q1 | 159 | 144 | 136 | TSQ4_Q3 | 154 | 145 | 128 |
| TSHS_1 | 37 | 46 | 32 | TSQ2_Q3 | 115 | 86 | 76 |
| TSQ1_Q2 | 90 | 90 | 62 | TSHS_2 | 99 | 90 | 44 |
| TSHS_2 | 76 | 69 | 66 | TSQ1_Q2 | 109 | 109 | 95 |
| TSQ2_Q3 | 166 | 133 | 92 | TSHS_1 | 85 | 88 | 74 |
| TSQ4_Q3 | 131 | 133 | 121 | TSQ0_Q1 | 88 | 84 | 78 |

**Table S4.** Key interatomic distances (Å) for TSQ0_Q1. Distances are reported for Li⁺, Na⁺, and K⁺ cations as M. M–O distances indicate interactions between the alkali cation and relevant framework oxygens, Si–O distances correspond to bonds within the reacting Si(OH)₄ monomer and framework, and H–O distances describe proton positions relevant to water formation during vacancy sealing, see Figure S4.

|  | **Li^+^ d(Å)** | **Na^+^ d(Å)** | **K^+^ d(Å)** |
| --- | --- | --- | --- |
| **M – O1** | 2.19 | 2.51 | 2.81 |
| **M – O2** | 2.07 | 2.43 | 2.89 |
| **M – O3** | 2.04 | 2.37 | 2.87 |
| **M – O4** | 3.54 | 3.94 | 4.35 |
| **M – O5** | 2.09 | 2.34 | 2.70 |
| **M – O6** | 3.37 | 3.70 | 4.20 |
| **M – O7** | 4.28 | 4.60 | 4.92 |
| **M – O8** | 4.15 | 4.44 | 4.86 |
| **M – O9** | 2.01 | 2.35 | 2.71 |
| **Si – O1** | 1.82 | 1.82 | 1.81 |
| **Si – O2** | 3.23 | 3.34 | 3.35 |
| **Si – O3** | 4.64 | 4.98 | 5.38 |
| **Si – O4** | 4.79 | 4.94 | 5.06 |
| **Si – O5** | 1.65 | 1.65 | 1.65 |
| **Si – O6** | 1.72 | 1.72 | 1.71 |
| **Si – O7** | 1.65 | 1.66 | 1.66 |
| **Si – O8** | 1.97 | 1.99 | 2.00 |
| **Si – O9** | 3.96 | 4.10 | 4.13 |
| **H1 – O8** | 1.19 | 1.20 | 1.20 |
| **H2 – O8** | 0.97 | 0.97 | 0.97 |
| **H1 – O5** | 1.28 | 1.26 | 1.26 |

**Table S5.** Key interatomic distances (Å) for TSHS_1. Distances are reported for Li⁺, Na⁺, and K⁺ cations as M. M–O distances indicate interactions between the alkali cation and relevant framework oxygens, Si–O distances correspond to bonds within the reacting Si(OH)₄ monomer and framework, and H–O distances describe proton positions relevant to the proton exchange between O2 and O5, see Figure S5.

|  | **Li^+^ d(Å)** | **Na^+^ d(Å)** | **K^+^ d(Å)** |
| --- | --- | --- | --- |
| **M – O1** | 3.69 | 3.98 | 4.53 |
| **M – O2** | 3.53 | 3.58 | 4.47 |
| **M – O3** | 5.70 | 5.86 | 6.73 |
| **M – O4** | 3.87 | 4.07 | 4.95 |
| **M – O5** | 2.11 | 2.41 | 2.73 |
| **M – O6** | 1.89 | 2.27 | 2.63 |
| **M – O7** | 4.01 | 4.29 | 4.30 |
| **M – O8** | 4.03 | 4.07 | 4.67 |
| **M – O9** | 1.93 | 2.33 | 2.71 |
| **Si – O1** | 1.66 | 1.65 | 1.65 |
| **Si – O2** | 3.85 | 3.86 | 3.89 |
| **Si – O3** | 5.00 | 4.97 | 4.98 |
| **Si – O4** | 4.02 | 4.02 | 4.05 |
| **Si – O5** | 1.60 | 1.60 | 1.60 |
| **Si – O6** | 1.68 | 1.67 | 1.67 |
| **Si – O7** | 1.65 | 1.66 | 1.65 |
| **Si – O8** | 3.19 | 3.25 | 3.27 |
| **Si – O9** | 4.23 | 4.89 | 5.15 |
| **H1 – O8** | 1.29 | 1.25 | 1.28 |
| **H2 – O8** | 1.07 | 1.11 | 1.13 |
| **H2 – O5** | 1.42 | 1.31 | 1.32 |
| **H1 – O2** | 1.16 | 1.19 | 1.14 |

**Table S6.** Key interatomic distances (Å) for TSQ1_Q2. Distances are reported for Li⁺, Na⁺, and K⁺ cations as M. M–O distances indicate interactions between the alkali cation and relevant framework oxygens, Si–O distances correspond to bonds within the reacting Si(OH)₄ monomer and framework, and H–O distances describe proton positions relevant to water formation during vacancy sealing, see Figure S6.

|  | **Li^+^ d(Å)** | **Na^+^ d(Å)** | **K^+^ d(Å)** |
| --- | --- | --- | --- |
| **M – O1** | 3.99 | 4.32 | 4.34 |
| **M – O2** | 3.08 | 3.11 | 3.80 |
| **M – O3** | 5.31 | 5.33 | 5.50 |
| **M – O4** | 5.96 | 6.08 | 6.62 |
| **M – O5** | 1.83 | 2.19 | 2.59 |
| **M – O6** | 4.53 | 4.69 | 5.34 |
| **M – O7** | 4.04 | 4.47 | 4.67 |
| **M – O8** | 1.90 | 2.27 | 2.74 |
| **M – O9** | 1.97 | 2.49 | 2.94 |
| **Si – O1** | 1.67 | 1.67 | 1.67 |
| **Si – O2** | 1.71 | 1.71 | 1.70 |
| **Si – O3** | 3.92 | 3.89 | 3.81 |
| **Si – O4** | 3.29 | 3.32 | 3.35 |
| **Si – O5** | 1.64 | 1.63 | 1.64 |
| **Si – O6** | 1.68 | 1.68 | 1.68 |
| **Si – O7** | 2.16 | 2.22 | 2.27 |
| **Si – O8** | 4.46 | 4.67 | 4.55 |
| **Si – O9** | 3.50 | 3.60 | 3.54 |
| **H1 – O7** | 1.24 | 1.27 | 1.32 |
| **H2 – O7** | 0.97 | 0.97 | 0.97 |
| **H1 – O5** | 1.21 | 1.17 | 1.15 |

**Table S7.** Key interatomic distances (Å) for TSHS_2. Distances are reported for Li⁺, Na⁺, and K⁺ cations as M. M–O distances indicate interactions between the alkali cation and relevant framework oxygens, Si–O distances correspond to bonds within the reacting Si(OH)₄ monomer and framework, and H–O distances describe proton positions relevant to the proton exchange between O3 and O5, see Figure S7.

|  | **Li^+^ d(Å)** | **Na^+^ d(Å)** | **K^+^ d(Å)** |
| --- | --- | --- | --- |
| **M – O1** | 3.58 | 3.46 | **3.22** |
| **M – O2** | 4.30 | 4.51 | 4.52 |
| **M – O3** | 4.06 | 4.06 | 3.72 |
| **M – O4** | 5.74 | 5.68 | 5.43 |
| **M – O5** | 1.89 | 2.25 | 2.69 |
| **M – O6** | 4.37 | 4.57 | 4.84 |
| **M – O7** | 2.84 | 2.99 | 2.81 |
| **M – O8** | 1.90 | 2.26 | 2.68 |
| **M – O9** | 4.06 | 4.32 | 4.40 |
| **Si – O1** | 1.64 | 1.65 | 1.66 |
| **Si – O2** | 1.64 | 1.65 | 1.65 |
| **Si – O3** | 3.49 | 3.51 | 3.57 |
| **Si – O4** | 3.72 | 3.65 | 3.66 |
| **Si – O5** | 1.61 | 1.61 | 1.60 |
| **Si – O6** | 1.64 | 1.65 | 1.65 |
| **Si – O7** | 3.13 | 3.15 | 3.16 |
| **Si – O8** | 4.86 | 5.22 | 5.70 |
| **Si – O9** | 5.81 | 6.02 | 6.07 |
| **H1 – O7** | 1.22 | **1.28** | 1.14 |
| **H2 – O7** | 1.17 | 1.17 | 1.43 |
| **H1 – O5** | 1.19 | 1.17 | 1.29 |
| **H2 – O3** | 1.28 | 1.24 | 1.08 |

**Table S8.** Key interatomic distances (Å) for TSQ2_Q3. Distances are reported for Li⁺, Na⁺, and K⁺ cations as M. M–O distances indicate interactions between the alkali cation and relevant framework oxygens, Si–O distances correspond to bonds within the reacting Si(OH)₄ monomer and framework, and H–O distances describe proton positions relevant to water formation during vacancy sealing, see Figure S8.

|  | **Li^+^ d(Å)** | **Na^+^ d(Å)** | **K^+^ d(Å)** |
| --- | --- | --- | --- |
| **M – O1** | 3.75 | 3.75 | 4.23 |
| **M – O2** | 4.67 | 5.01 | 5.44 |
| **M – O3** | 3.93 | 3.88 | 4.11 |
| **M – O4** | 6.60 | 6.72 | 7.16 |
| **M – O5** | 1.87 | 2.30 | 2.68 |
| **M – O6** | 3.37 | 3.81 | 4.28 |
| **M – O7** | 6.16 | 6.57 | 7.00 |
| **M – O8** | 1.89 | 2.27 | 2.72 |
| **M – O9** | 3.20 | 3.18 | 2.77 |
| **Si – O1** | 1.67 | 1.66 | 1.66 |
| **Si – O2** | 1.67 | 1.69 | 1.70 |
| **Si – O3** | 1.72 | 1.73 | 1.74 |
| **Si – O4** | 3.47 | 3.56 | 3.55 |
| **Si – O5** | 1.69 | 1.67 | 1.67 |
| **Si – O6** | 1.93 | 1.92 | 1.92 |
| **Si – O7** | 3.95 | 4.01 | 4.03 |
| **Si – O8** | 4.56 | 5.01 | 5.96 |
| **Si – O9** | 3.78 | 3.88 | 3.95 |
| **H1 – O6** | 1.26 | 1.22 | 1.22 |
| **H2 – O6** | 0.99 | 0.99 | 0.99 |
| **H1 – O5** | 1.21 | 1.25 | 1.25 |

**Table S9.** Key interatomic distances (Å) for TSQ1_Q2. Distances are reported for Li⁺, Na⁺, and K⁺ cations as M. M–O distances indicate interactions between the alkali cation and relevant framework oxygens, Si–O distances correspond to bonds within the reacting Si(OH)₄ monomer and framework, and H–O distances describe proton positions relevant to water formation during vacancy sealing, see Figure S9.

|  | **Li^+^ d(Å)** | **Na^+^ d(Å)** | **K^+^ d(Å)** |
| --- | --- | --- | --- |
| **M – O1** | 2.76 | 2.98 | 3.37 |
| **M – O2** | 3.67 | 3.73 | 3.28 |
| **M – O3** | 4.62 | 4.85 | 5.04 |
| **M – O4** | 4.49 | 4.73 | 4.76 |
| **M – O5** | 1.95 | 2.26 | 2.72 |
| **M – O6** | 3.94 | 3.84 | 3.37 |
| **M – O7** | 6.22 | 6.06 | 5.70 |
| **M – O8** | 1.93 | 2.30 | 2.68 |
| **M – O9** | 2.02 | 2.40 | 3.03 |
| **Si – O1** | 1.76 | 1.74 | 1.72 |
| **Si – O2** | 1.66 | 1.66 | 1.66 |
| **Si – O3** | 1.71 | 1.69 | 1.67 |
| **Si – O4** | 2.15 | 2.26 | 2.35 |
| **Si – O5** | 1.64 | 1.63 | 1.62 |
| **Si – O6** | 3.26 | 3.23 | 3.18 |
| **Si – O7** | 5.83 | 5.82 | 5.80 |
| **Si – O8** | 4.79 | 5.43 | 6.00 |
| **Si – O9** | 3.64 | 4.46 | 5.08 |
| **H1 – O4** | 1.15 | 1.18 | 1.18 |
| **H1 – O6** | 1.04 | 1.07 | 1.14 |
| **H2 – O5** | 1.54 | 1.42 | 1.29 |
| **H2 – O6** | 1.28 | 1.26 | 1.27 |

**References**

[1]	A. Burton, E. Terefenko, H. Wang, M. Paccagnini, A. Sattler, *Microporous and Mesoporous Materials* **2023**, *350*, 112411.

[2]	S. Sharma, S. R. G. Balestra, R. Baur, U. Agarwal, E. Zuidema, M. S. Rigutto, S. Calero, T. J. H. Vlugt, D. Dubbeldam, *Mol. Simul.* **2023**, *49*, 893.

[3]	F. N. Fritsch, R. E. Carlson, *SIAM J. Numer. Anal.* **1980**, *17*, 238.

[4]	F. N. Fritsch, J. Butland, *SIAM Journal on Scientific and Statistical Computing* **1984**, *5*, 300.
